# Post-COVID-19 condition and disparities in daily functional activities in England: a retrospective analysis of data from the Virus Watch community cohort

**DOI:** 10.1016/j.lanprc.2025.100093

**Published:** 2026-01

**Authors:** Wing Lam Erica Fong, Sarah Beale, Vincent Grigori Nguyen, Jana Kovar, Alexei Yavlinsky, Andrew C Hayward, Ibrahim Abubakar, Sander M J van Kuijk, Robert W Aldridge

**Affiliations:** a Institute of Health Informatics, University College London, London, UK; b Institute of Epidemiology and Health Care, University College London, London, UK; c Faculty of Population Health Sciences, University College London, London, UK; d Department of Clinical Epidemiology and Medical Technology Assessment, Maastricht University Medical Centre (MUMC), Maastricht, Netherlands; e Department of Population, Policy and Practice, UCL Great Ormond Street Institute of Child Health, London, UK; f National Institute for Health and Care Research (NIHR) Health Protection Research Unit in Health Analytics and Modelling, Department of Infectious Disease Epidemiology, School of Public Health, Imperial College London, London, UK; g The Institute for Health Metrics and Evaluation, University of Washington, Seattle, WA, USA

## Abstract

**Background:**

Post-COVID-19 condition is increasingly recognised to impair daily functioning. In the UK and many other settings worldwide, primary care is the first point of contact and key provider of care for people with the condition. Consequently, identifying groups at heightened risk allows primary care services to tailor assessment, support, and referral pathways more effectively. This analysis aims to investigate the role of socioeconomic deprivation, migration status, and ethnicity in experiencing limitations in six functional activities.

**Methods:**

We used data from Virus Watch, a prospective community cohort study in England and Wales, which enrolled 58 628 participants between June 22, 2020, and March 31, 2025. Participants self-reported new persistent symptoms and their effects on six functional activities (work or education, concentration, self-care, care for others, performance of necessary activities outside the house, and engagement in enjoyable activities) through six surveys over 4 years (2021–24). In this retrospective analysis, participants were included if they (1) were aged 18 years or older and lived in England; (2) were successfully linked to national hospitalisation, COVID-19 testing and vaccination, and mortality data; (3) were not admitted to hospital for or with COVID-19; and (4) self-reported persistent symptoms that met the WHO consensus definition of post-COVID-19 condition. Logistic regression was used to assess how deprivation level, migration status, and ethnic minority status were associated with the odds of experiencing each functional limitation, adjusting for sociodemographic variables.

**Findings:**

776 individuals with post-COVID-19 condition were included in this analysis (552 [71%] female, 224 [29%] male; 685 [88%] White British, 88 [11%] ethnic minority, and 11 [1%] missing ethnicity). 686 (88%) reported experiencing limitations in at least one daily activity. Individuals with post-COVID-19 condition in Index of Multiple Deprivation quintile (IMD) 1 (the most deprived quintile) had higher adjusted odds of limitations in work or education (adjusted odds ratio [aOR]; 2·30 [95% CI 1·12–4·93]), concentration (2·78 [1·42–5·81]), self-care (2·11 [1·06–4·16]), and doing necessary activities outside the house (2·06 [1·12–3·90]) than those in IMD 5 (the least deprived). Those in IMD 2 also experienced increased odds of limitations in work or education (1·90 [1·09–3·34]) and concentration (1·91 [1·17–3·14]) compared to those in IMD 5. We found no evidence of associations between migration status or minority ethnicity status with functional limitations (migration status aOR range from 0·67 [0·25–1·73] to 1·52 [0·64–3·77]; minority ethnicity status aOR range from 0·66 [0·35–1·17] to 1·41 [0·82–2·53]).

**Interpretation:**

Socioeconomic deprivation, rather than migration status or ethnicity, primarily drives functional limitations within this cohort of individuals with post-COVID-19 condition. Functional limitations might perpetuate cycles of deprivation and further exacerbate health inequalities. Equitable access to primary care and rehabilitation and support services, alongside workplace and educational adaptations, is needed to address the functional limitations of those affected by post-COVID-19 condition. Similar strategies might also be used to support individuals with long-term functional impairment following other post-acute infection syndromes.

**Funding:**

Medical Research Council, Wellcome Trust, and European Union.

## Introduction

Post-COVID-19 condition, or long COVID, is defined as the presence of new or persistent symptoms lasting more than 2 months and occurring within 3 months of an acute SARS-CoV-2 infection, without an alternative diagnosis.^1^ These persistent symptoms, most commonly fatigue, shortness of breath, and cognitive dysfunction, can impact an individual’s ability to carry out everyday activities, such as attending work or education, and caring for themselves and others.^2^^,^^3^ Increasing evidence shows that post-COVID-19 condition impairs daily functioning, particularly with work ability, cognitive function, and self-care. Longitudinal cohort studies and systematic reviews suggest that a significant proportion of people with post-COVID-19 condition experience a decline in functional abilities months after infection.^4^, ^5^, ^6^, ^7^ One meta-analysis reported that, across multiple countries, 13–55% of individuals with post-COVID-19 condition were unable to return to work up to 12 months after infection, with many also experiencing increased absenteeism and reduced productivity.^4^ Cognitive dysfunction and memory issues are frequently reported in people with post-COVID-19 condition, impacting their ability to concentrate, process information, and perform complex tasks.^2^ Beyond occupational and cognitive limitations, post-COVID-19 condition is also associated with impairments in activities of daily living, including both basic self-care tasks and instrumental activities of daily living, such as managing household tasks, preparing meals, and shopping.^5^^,^^8^ These impairments can in turn reduce independence and quality of life.^5^^,^^8^

**Research in context**

**Evidence before this study**

We searched PubMed and Web of Science for articles in English indexed from March 1, 2020, up to May 31, 2025, on the association between deprivation, migration status, and ethnicity, and functional limitations of post-COVID-19 condition. The search string combined terms for post-COVID-19 condition (eg, “post-COVID condition”, “long COVID”), social and demographic factors (eg, “socioeconomic status”, “migration status”, “ethnicity”), and functional outcomes (eg, “functional impairment”, “work ability”, “cognitive impairment”, “activities of daily living”). The search identified several US-based studies reporting greater functional limitations among Black and Hispanic populations than White populations, but few data exist outside the USA, and none addressed deprivation and migration status. We therefore excluded the search terms related to social determinants in a follow-up search to focus on the broader literature on functional limitations. Most studies focused on hospitalised populations and often lacked detail on specific functional activities. One UK-based study reported greater functional impairment, measured using the Work and Social Adjustment Scale, in individuals in the most deprived quintile than in those in less deprived quintiles in the UK. However, the impact of migration status remains largely unexplored.

**Added value of this study**

To our knowledge, this is the first study to examine how deprivation, migration status, and ethnic minority status influence specific functional limitations among adults with post-COVID-19 condition in England. By disaggregating specific daily activities, we found that individuals living in more deprived areas experienced greater odds of limitations in work or education attendance or participation, concentration, self-care, and performance of necessary activities outside the house than did those living in the least deprived areas. By contrast, no disparities were observed for migrants or ethnic minorities. Our findings provide granular insights into the role of social determinants, particularly deprivation, in influencing the functional impacts of post-COVID-19 condition. These insights could then allow the development of more targeted interventions and policy responses to support those most affected.

**Implications of all the available evidence**

Alongside existing evidence, our findings highlight the need for targeted interventions to address the disproportionate impact of post-COVID-19 condition on individuals in deprived communities. Policy responses should prioritise equitable access to primary care, rehabilitation and support services through adapted referral pathways and culturally appropriate outreach. Reforming statutory sick pay eligibility and ensuring the availability of flexible workplace or educational arrangements should also be ensured for those affected. Addressing these systemic barriers will prevent further widening of health inequalities and support a more equitable recovery from the long-term effects of COVID-19. It could also inform current and future public health strategies for post-acute infection syndromes, where similar socioeconomic and structural factors might influence recovery.

Socioeconomically deprived populations, migrants, and ethnic minority groups in the UK were disproportionately affected during the COVID-19 pandemic, with higher infection rates, greater disease severity, and increased risk of developing post-COVID-19 condition.^9^ These groups are often interrelated and overlap, as migrants and ethnic minority groups are more likely to live in deprived areas and experience increased exposure risk, differential access to care, and underlying adverse social determinants of health.^10^ Although post-COVID-19 condition can lead to substantial functional limitations in affected individuals across hospitalised and general populations, there is little research on how socioeconomic deprivation, ethnicity, and migration status influence the functional outcomes of post-COVID-19 condition. Emerging evidence suggests that individuals who are socioeconomically deprived might be at increased risk of experiencing functional limitations as a result of post-COVID-19 condition. For example, a UK-based study found that individuals in the most deprived quintile had higher levels of functional impairment, as measured by the Work and Social Adjustment Scale, compared with those in less deprived quintiles.^11^ Similarly, several US-based studies observed greater cognitive impairments, including difficulty concentrating, among Black and Hispanic individuals hospitalised for COVID-19, but few data exist outside the USA.^12^^,^^13^ Overall, the evidence for functional limitations among these populations is scarce, but the increased risk of functional limitations, particularly reduced work, educational, and self-care abilities, from post-COVID-19 condition might reinforce and exacerbate existing health inequalities. Understanding these disparities is essential to inform equitable health-care access, guide targeted rehabilitation and support services, and shape policy interventions to reduce the long-term impact of COVID-19 in these communities. In the UK and many other settings worldwide, primary care plays a central role in identifying and supporting people with persistent symptoms. Evidence on inequalities is crucial for allowing primary care teams to tailor care pathways and ensure timely access to appropriate services.

Thus, this retrospective study used data from Virus Watch, a community cohort study, to investigate how deprivation, migration status, and ethnicity are associated with experiencing six specific functional limitations in daily life: attending or participating in work or education, concentrating, maintaining self-care, caring for others, performing necessary activities outside the home, and engaging in enjoyable activities. This study aims to generate evidence on social inequalities in post-COVID-19 outcomes to inform equitable primary care, public health, and policy responses.

## Methods

### Study design and participants

Virus Watch was a large prospective community cohort study conducted from June 22, 2020, to March 31, 2025, to examine the transmission and impact of COVID-19 in England and Wales (n=58 628). The study recruited entire households through social media, SMS, and personalised postal campaigns supported by general practices. To increase the engagement of ethnic minority participants, targeted recruitment using postal letters was conducted via 90 general practice clinics in nine local clinical research networks. The general practice clinics facilitated the distribution of these invitations but did not actively encourage participation. Culturally tailored materials (participant information sheets and consent forms translated into nine languages) and a £20 household incentive were used to support participation. Participants self-selected into the study through voluntary registration, with eligibility limited to households with internet access and a lead household member proficient in reading English, although consent forms were available in multiple languages. Participants completed weekly online surveys on acute COVID-19 symptoms, testing, and vaccination, along with occasional in-depth questionnaires on COVID-19-related topics such as behavioural practices, health-care access, and long-term symptoms. Furthermore, the Virus Watch cohort was linked to Hospital Episode Statistics (HES) Admitted Patient Care, which contains details of all admissions at National Health Service (NHS) hospitals in England and admissions to private or charitable hospitals paid for by the NHS.^14^ The Virus Watch dataset was also linked to the national COVID-19 testing data (Second Generation Surveillance System [SGSS]), COVID-19 vaccination data (the UK National Immunisation Management Service [NIMS]), and mortality data from the Office for National Statistics (ONS). The full study design, methodology, and the consistency of participants’ responses to surveys are described in the study protocol and cohort profile papers.^15^^,^^16^

**Figure 1:**
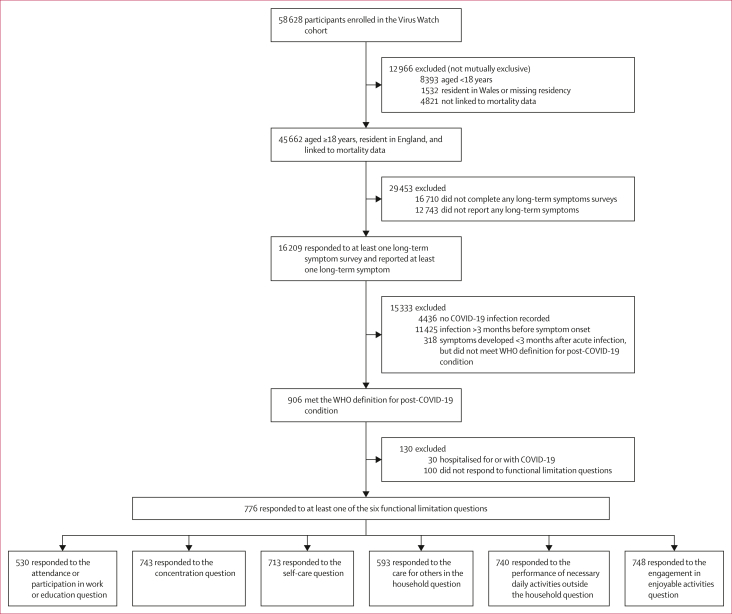
Cohort flow diagram

Participants in this retrospective analysis were a subset of the Virus Watch study cohort (figure 1). They were included if they (1) were aged 18 years or older and registered with an English postcode; (2) were successfully linked to either HES, NIMS, SGSS, or ONS mortality data; (3) were not admitted to hospital for or with COVID-19; and (4) self-reported persistent symptoms that met the WHO consensus definition of post-COVID-19 condition (n=776; figure 1). Hospitalised individuals were excluded to prevent misclassifying cases of post-intensive-care or post-sepsis syndromes as post-COVID-19 condition and to allow us to focus on community-acquired post-COVID-19 condition. Those younger than 18 years or residing in Wales were excluded due to the lack of linked hospitalisation data for these groups.

Cases of post-COVID-19 condition (n=906) were identified through six surveys on long-term symptoms administered across 4 years (Feb 17, 2021; May 26, 2021; March 22, 2022; Feb 28, 2023; Oct 10, 2023; April 16, 2024). In each long-term symptom survey, participants reported the development of any new symptoms lasting at least 4 weeks since the last survey (or since February, 2020 for the first two long-term symptom surveys), irrespective of whether the symptoms were attributable to COVID-19 or post-COVID-19 condition, the onset dates of their three most severe symptoms, and whether they were ongoing or resolved (appendix pp 5–6). Symptom duration was calculated from symptom onset to survey completion (for ongoing symptoms) or reported resolution date. Following the WHO definition, post-COVID-19 condition was defined as occurring in individuals with persistent symptoms lasting at least 2 months that developed within 3 months of a confirmed SARS-CoV-2 infection, unexplained by another diagnosis (appendix p 7 shows sources of SARS-CoV-2 infection, and appendix pp 64–74 shows details on the long-term infection survey).^1^ Although data were collected from Feb 1, 2020, to April 30, 2024, only cases reported up to March 31, 2024 were included, because hospitalisation data were limited to this period.

Virus Watch was approved by the Hampstead NHS Health Research Authority Ethics Committee (20/HRA/2320) and conformed to the ethics standards set out in the Declaration of Helsinki. Participants provided informed written consent for all aspects of the study. This retrospective analysis falls within the scope of the original consent. Therefore, no additional permission to analyse the data was required.

### Exposures

We examined three exposures: socioeconomic deprivation, migration status, and ethnic minority status.

Socioeconomic deprivation was measured using quintiles of the Index of Multiple Deprivation (IMD).^17^ IMD is calculated for small local areas in England and typically covers seven dimensions of deprivation: crime, employment, education, income, health, living environment, and barriers to housing and services. These areas are ranked from most to least deprived relative to others and categorised into five quintiles. The first quintile (IMD 1) represents the most deprived areas, and the fifth quintile (IMD 5) represents the least deprived.^17^ The IMD classification for each participant was determined based on their self-reported residential postcode in the baseline survey, which was linked to the May 2020 ONS Postcode Lookup dataset.^18^ For this analysis, IMD 5 was the reference category.

Migration status was determined by self-reported country of birth, with individuals born outside the UK classified as migrants. The UK-born group served as the reference category.

Ethnic minority status was determined using self-reported ethnicity. Participants identifying as White Irish, White other, mixed, South Asian, other Asian, Black, or other were categorised as ethnic minorities.^19^ The White British group was used as the reference category.

### Outcomes

In each long-term symptom survey, only participants who reported experiencing any new persistent symptoms were asked how these symptoms affected their ability to: (1) go to or participate in work or education; (2) concentrate on things (eg, reading, watching TV); (3) take care of themselves (eg, wash, dress, and feed themselves); (4) take care of others in the household; (5) do necessary daily activities outside the household; and (6) do activities that they enjoy (eg, hobbies). Participants were identified to have a specific functional limitation if they answered “Yes, a lot” or “Yes, a little”. Those reporting “Not at all” indicated they did not experience the specific functional limitation, while those reporting “Not applicable” were excluded from the analysis for that functional limitation. If a participant responded to more than one survey on persistent symptoms, we included only the earliest response that met the definition of post-COVID-19 condition in the analysis, because later survey responses could not be distinguished as unresolved post-COVID-19 condition or a new episode of post-COVID-19 condition or another post-acute infection syndrome.

### Covariates

The covariates considered were age group (0–24 years, 25–44 years, 45–64 years, ≥65 years), sex assigned at birth (male and female), and pre-infection health. Sex was primarily self-reported by participants. Where sex was missing, we used the value recorded in linked national data. For participants with missing information in both sources, sex was inferred using a validated name-based dictionary.^20^ This applied to only ten individuals within the analysis cohort. Pre-infection health was assessed based on self-reported health conditions at baseline, which we then used to classify whether participants were “not clinically vulnerable”, “clinically vulnerable”, or “extremely clinically vulnerable”, according to the NHS and UK Government criteria for clinical vulnerability.^21^

### Statistical analysis

Due to the retrospective nature of this analysis, no formal sample size calculations were done. Baseline demographic and clinical characteristics were summarised using descriptive statistics. We used logistic regression to investigate how deprivation, migration status, and ethnic minority status affected the odds of experiencing each of the six functional limitations as a result of post-COVID-19 condition. Results are expressed as odds ratios (ORs) with 95% CIs. Separate models were fitted for each exposure and functional limitation outcome. For models where migration status or ethnic minority status was the exposure, we conducted complete case analyses. However, for models where IMD was the exposure, participants with missing migration status were kept by including them in a separate “Missing” category, due to the high proportion of missingness (23·3%) for this variable.

We performed three sensitivity analyses. First, we conducted complete case analyses for models with IMD as the exposure to compare results with those from the main analysis that included those with missing migration status. Second, we recalculated the IMD quintile measure, which excludes the health domain when calculating the ranking score, using the methodology described by Adams and White.^22^ We used the IMD quintile measure excluding the health domain as the exposure to assess whether the inclusion of the health domain introduced endogeneity bias. Lastly, for analyses with migration status as the exposure, we included participants with missing country of birth in a separate “missing” category.

Potential confounders for each exposure were identified using directed acyclic graphs (DAGs; appendix pp 8–13). Multicollinearity was assessed (appendix pp 18–19). For deprivation, the minimal adjustment set included age, sex, ethnic minority status, and migration status. For migration status, the set consisted of age, sex, and ethnic minority status. For ethnic minority status, no confounders were identified, but we adjusted for age and sex based on a-priori considerations. Vaccination status, acute infection severity, and symptom duration and frequency of post-COVID-19 condition were considered because of their influence on experiencing functional limitations. However, they were not included in the adjustment sets since they were deemed as mediator variables in the DAGs. In our secondary analysis, we additionally adjusted for pre-infection health in models with deprivation and migration status as exposures, reflecting alternative assumptions about the directionality between pre-infection health and the exposures—ie, reversing the direction of the relationship between pre-infection health and deprivation or migration status. We did not apply formal adjustments for multiple testing, as the analyses were based on pre-specified, related exposures and outcomes rather than broad exploratory screening.

We followed the STROBE guidelines for reporting the study (appendix pp 3–4).

Survey response data were extracted from REDCap, linked, and analysed in R (version 4.4.1) and RStudio (version 2025.05.1+513; Posit).

### Role of the funding source

The funders of the study had no role in study design, data collection, data analysis, data interpretation, or writing of the report.

## Results

Of 58 628 participants enrolled in Virus Watch, 45 662 were aged 18 years and older, resident in England, and linked to HES, NIMS, SGSS, or ONS mortality data (figure 1). Among these, 28 952 participants responded to at least one long-term symptoms survey. We identified 906 participants who met the WHO criteria for post-COVID-19 condition. 30 (3%) of 906 participants were excluded due to hospitalisation for or with COVID-19, and 100 (11%) were excluded for missing functional limitations data. Among the remaining 776 individuals who completed the functional limitations section of the survey, 686 (88%) reported experiencing at least one functional limitation related to post-COVID-19 condition. The median time between the onset of post-COVID-19 condition and the reporting of functional limitations was 6·0 months (IQR 3·7–10·4).

Table 1 reports the sociodemographic and clinical characteristics of the analysis cohort. The majority within the cohort were aged 45–64 years (378 participants, 49%), female (552 participants, 71%), living in the least deprived neighbourhoods (186 participants, 24%), UK-born (538 participants, 69%), White British (685 participants, 88%), and not clinically vulnerable (380 participants, 49%). Cohort characteristics by exposures and outcomes are presented in the appendix (pp 13–15). High proportions of participants reported limitations in attending or participating in work or education (325 participants, 61%), concentrating (495 participants, 67%), performing daily activities outside the household (448 participants, 61%), and engaging in enjoyable activities (545 participants, 73%; table 2). Similar patterns were observed in both men and women (appendix p 17).

**Table 1:** Sociodemographic and clinical characteristics of the analysis cohort compared with the entire Virus Watch study cohort

	Virus Watch adult cohort in England (n=48 887)	Analysis cohort (n=776)	Population of England from ONS
**Age group**			
18–44 years	13 920 (28%)	93 (12%)	35%
45–64 years	19 097 (39%)	378 (49%)	26%
≥65 years	15 870 (32%)	305 (39%)	19%
**Sex**			
Male	21 449 (44%)	224 (29%)	49%
Female	26 826 (55%)	552 (71%)	51%
Intersex	60 (<1%)	··	··
Missing	552 (1%)	··	··
**IMD quintile**			
1 (most deprived)	4904 (10%)	68 (9%)	20%
2	7647 (16%)	139 (18%)	20%
3	9174 (19%)	165 (21%)	20%
4	11 027 (23%)	188 (24%)	20%
5 (least deprived)	12 459 (25%)	207 (27%)	20%
Missing	3676 (8%)	9 (1%)	··
**IMD quintile (excluding the health domain)**			
1 (most deprived)	4845 (10%)	66 (9%)	··
2	7905 (16%)	141 (18%)	··
3	9755 (20%)	183 (24%)	··
4	11 093 (23%)	191 (25%)	··
5 (least deprived)	11 613 (24%)	186 (24%)	··
Missing	3676 (8%)	9 (1%)	··
**Migration status**			
UK-born	28 298 (58%)	538 (69%)	83%
Not UK-born	5022 (10%)	57 (7%)	17%
Missing	15 567 (32%)	181 (23%)	··
**Ethnic minority status**			
White British	34 991 (72%)	685 (88%)	74%
White Irish or White other	3133 (6%)	48 (6%)	7%
Asian	2603 (5%)	15 (2%)	10%
Black, mixed, or other∗	1242 (3%)	17 (2%)	9%
Missing or prefer not to say	6918 (14%)	11 (1%)	··
**Clinical vulnerability**			
Not clinically vulnerable	23 575 (48%)	380 (49%)	··
Clinically vulnerable	12 047 (25%)	251 (32%)	··
Clinically extremely vulnerable	3476 (7%)	127 (16%)	··
Missing	9789 (20%)	18 (2%)	··

IMD=Index of Multiple Deprivation. ONS=Office for National Statistics.

∗ “Black” ethnic group combined with the “mixed” and “other” groups due to small cell count to avoid disclosure.

**Table 2:** Number and proportion of participants who reported the presence or absence of limitations in the ability to perform each daily functional activity

	Attendance or participation in work or education (n=530)	Concentration on things (n=743)	Self-care (n=713)	Care for others in the household (n=593)	Performance of daily activities outside the household (n=740)	Engagement in enjoyable activities (n=748)
Presence of limitation	325 (61%)	495 (67%)	186 (26%)	198 (33%)	448 (61%)	545 (73%)
Absence of limitation	205 (39%)	248 (33%)	527 (74%)	395 (67%)	292 (39%)	203 (27%)

**Figure 2:**
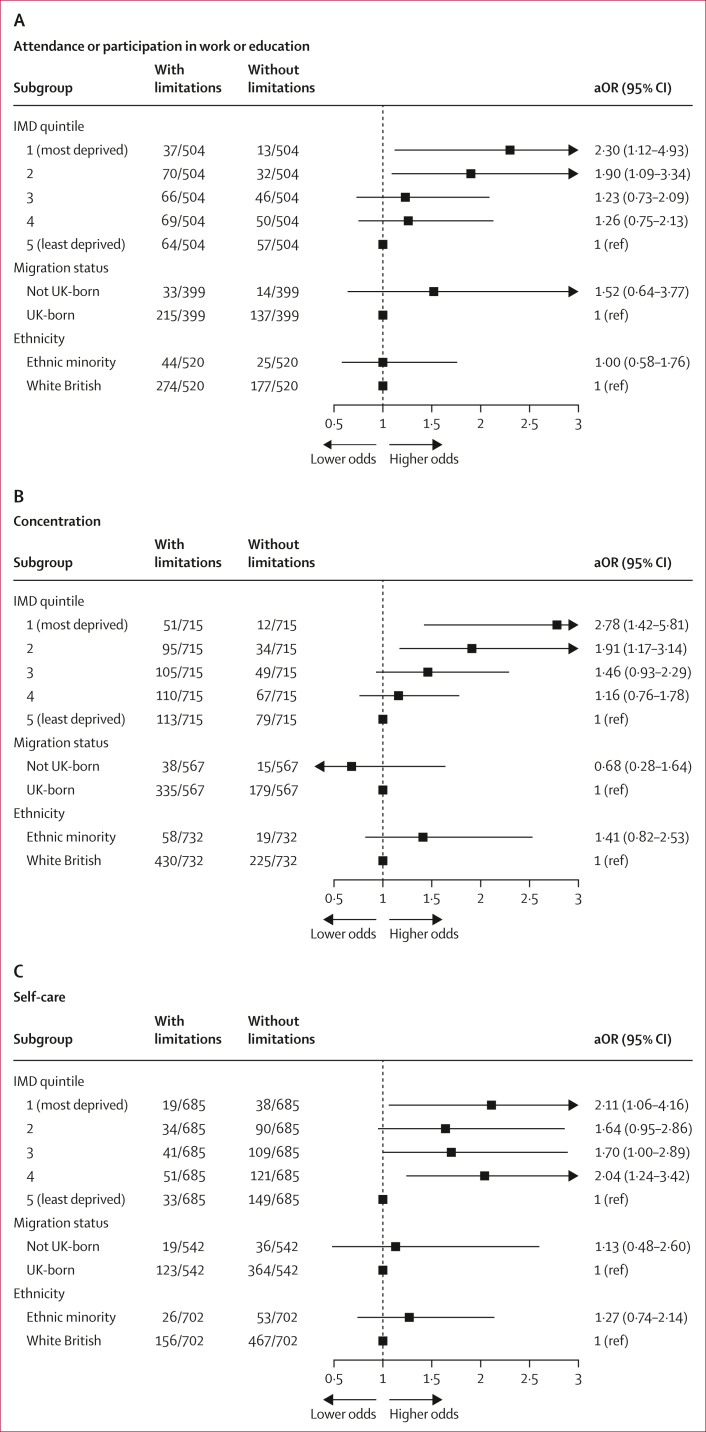

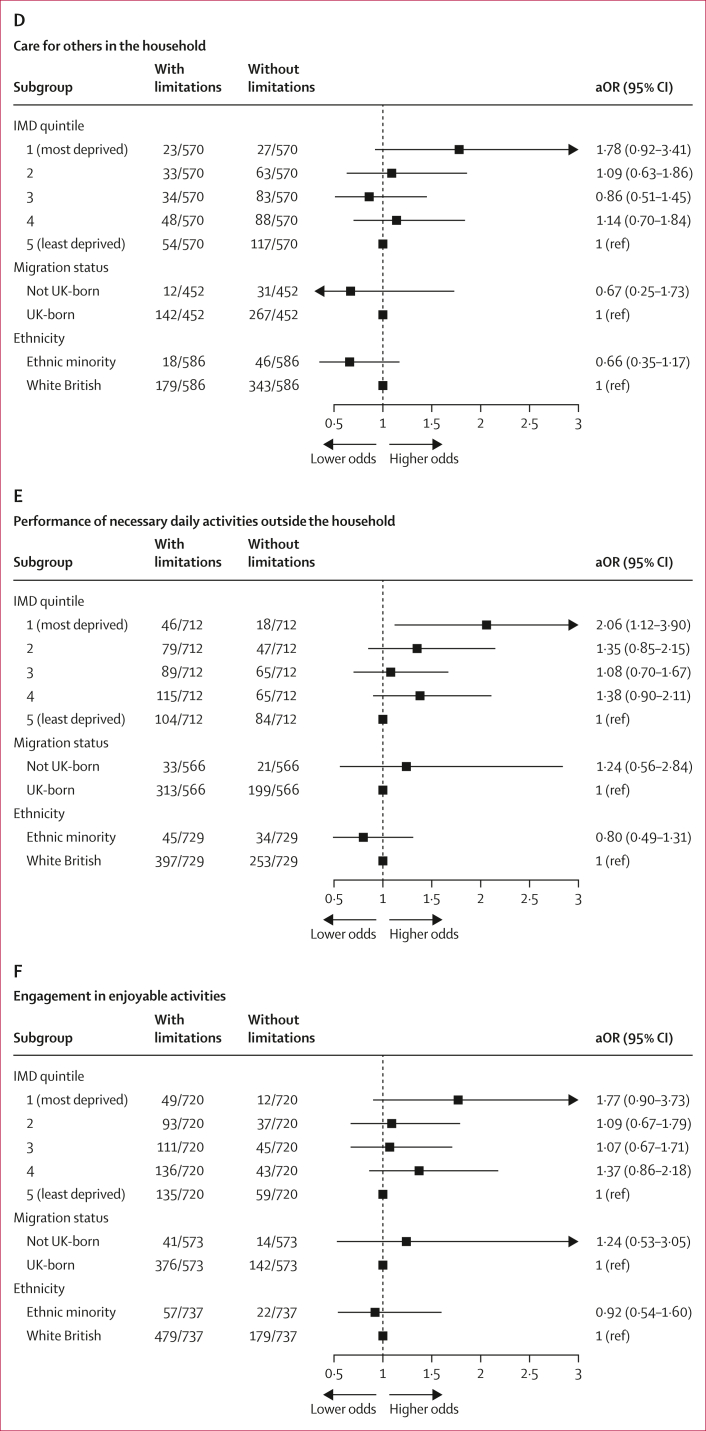
Association between deprivation, migration status, and ethnic minority status with the presence of functional limitations in participants with post-COVID-19 condition Logistic regression analysis for the association between deprivation, migration status, and ethnic minority status and six daily activities, including (A) attendance or participation in work or education, (B) concentration, (C) self-care, (D) care for others, (E) performance of necessary activities outside the house, and (F) engagement in enjoyable activities. Models with deprivation as the exposure were adjusted for age, sex, ethnic minority status, and migration status. Models with migration status as the exposure were adjusted for age, sex, and ethnic minority status, while models with ethnic minority status as the exposure were adjusted for age and sex. IMD=Index of Multiple Deprivation. aOR=adjusted odds ratio.

Compared with participants in the least deprived (IMD 5) group, those in the most deprived IMD quintiles (1 and 2) had increased odds of experiencing limitations, after adjusting for age, sex, ethnic minority status, and migration status. Specifically, individuals in the most deprived quintile (IMD 1) had increased odds of limitations in attending or participating in work or education (OR 2·30 [95% CI 1·12–4·93]) and in concentrating (2·78 [1·42–5·81]; figure 2A, B, appendix pp 20–22). Participants in IMD 2 also had higher odds in these domains (work or education: 1·90 [1·09–3·34]; concentration: 1·91 [1·17–3·14]) than those in IMD 5. Participants in the most deprived areas also had greater odds of self-care limitations (2·11 [1·06–4·16]) and of difficulties performing necessary activities outside the house (2·06 [1·12–3·90]) than those in the least deprived group (figure 2C, E, appendix pp 23, 25). Even though the point estimates pointed towards higher odds of limitations in caring for others (1·78 [0·92–3·41]) and in engaging in enjoyable activities (1·77 [0·90–3·73]) among participants in the most deprived areas than in those in the least deprived areas, the 95% CI overlapped 1 (figure 2D, F, appendix pp 24, 27).

The associations between area-level deprivation (IMD) and limitations in work or education attendance or participation and in concentration persisted after further adjusting for pre-infection health status (appendix pp 20–28). However, the effect of area-level deprivation on self-care activities, caring responsibilities, and engagement in enjoyable activities dropped.

In the complete case sensitivity analysis, most associations were attenuated, with wider confidence intervals that included 1, except for the association with limitations in concentration, which persisted (OR 2·89 [1·35–6·79]; appendix pp 42–49). However, the direction of associations remained consistent across all outcomes. In the sensitivity analysis using IMD quintile excluding the health domain, associations remained consistent with those of the main analysis (appendix pp 49–57).

No association was observed between migration status and experiencing limitations in any functional activities after accounting for age, sex, and minority ethnic status (figure 2A–F; appendix pp 29–35). This finding persisted following further adjustment for pre-infection health status and also in the sensitivity analyses, which included participants with unknown country of birth under “Missing” (appendix pp 58–63).

Similarly, after adjusting for age and sex, our findings indicated a lack of evidence for a difference in the odds of experiencing functional limitations between White British and ethnic minority participants (appendix pp 38–43).

## Discussion

Using data from a community cohort in England, we observed that people living in more deprived areas had increased odds of experiencing functional limitations related to post-COVID-19 condition, specifically attending or participating in work or education, concentrating, maintaining self-care, and doing daily activities outside the household, compared with those in the least deprived areas. In contrast, migrants and non-migrants, and ethnic minority and White British participants showed similar odds of experiencing functional limitations. These findings indicate that socioeconomic deprivation, rather than migration status or ethnicity, primarily drives functional limitations within this cohort.

Our findings are consistent with previous literature, indicating a greater likelihood of experiencing limitations in attending work or education due to post-COVID-19 condition among individuals living in more deprived areas than those in the least deprived areas.^11^ This finding aligns with the findings from Davies and colleagues, who reported a higher risk of limitations in activities of daily living among individuals aged 50 years or older living in more deprived areas than in those living in the least deprived areas, irrespective of post-COVID-19 condition.^23^ Similarly, our results regarding migration status and experiencing functional limitations broadly align with those from a South London community study, which reported increased odds of functional limitations due to poor mental health among migrants compared with non-migrants, but no difference for functional limitations due to physical health problems.^24^ Although their study examined general limitations rather than limitations associated with post-COVID-19 condition, it suggests that migration status alone might not be strongly associated with physical health-related functional limitations. Furthermore, we observed some evidence of an association between ethnic minority status and limitations in concentrating, a relationship previously reported by multiple US-based studies, highlighting the need for further research in a larger cohort, as our findings might reflect the small number of ethnic minority participants rather than a true lack of association.^12^^,^^13^

Deprived communities might face an increased likelihood of experiencing functional limitations because of systemic barriers. Reduced access to rehabilitation services is a key structural barrier, with evidence suggesting that people from socioeconomically deprived areas were less likely to be referred to services related to post-COVID-19 condition.^25^ Primary care plays a central part in recognising persistent symptoms and initiating referrals. However, shorter consultation rates, limited continuity of care, and lower primary care capacity in more deprived areas might further reduce access to timely assessment and referral. Barriers, such as lack of awareness, fear of stigma, and distrust of health-care providers, might further reduce service uptake in these populations. Furthermore, people with long-term conditions who are unable to access rehabilitation services are less likely to attend or participate in work or education.^26^ While some employers and educational institutions offer more flexible arrangements for post-COVID-19 condition, those in low-paid, insecure jobs lack such options, including access to flexible hours, extended rest breaks, remote work or learning options, or phased returns. Additionally, statutory sick pay in the UK is only available to employees earning above the income threshold, meaning that inadequate sick pay might compel individuals with post-COVID-19 condition to return to work despite feeling unwell.^27^

These structural barriers make it difficult for affected individuals to manage persistent symptoms and remain in, or return to, work or education, contributing to prolonged absences and reduced participation. These limitations can have far-reaching consequences, including financial insecurity, reduced quality of life, and poorer mental health, which might further impact daily functioning.^28^ Ultimately, disruptions to employment or education could perpetuate cycles of deprivation and exacerbate existing health inequalities, underscoring the need to address these systemic barriers to support recovery and minimise the broader social and economic impacts of post-COVID-19 condition.

Policy responses should prioritise equitable, targeted rehabilitation services, such as adapted referral pathways, extended hours, and community outreach, that will address the full spectrum of limitations related to post-COVID-19 condition.^29^ Increasing primary care capacity, particularly in deprived areas, through increased appointment availability, longer consultations for complex cases, and improved integration with community rehabilitation services, could allow early identification and ongoing monitoring of post-COVID-19 condition and related functional limitations, and more equitable referral into support services. Culturally appropriate public health campaigns are also essential to raise awareness and reduce stigma around post-COVID-19 condition and promote the availability of rehabilitation services. Additionally, workplace and educational institutions should provide flexible arrangements, including remote options and occupational health support, to maintain participation from those affected. Educating employers about this condition can facilitate better workplace adaptations to improve job and education retention. Statutory sick pay reform is also essential to ensure adequate financial support for workers in low-paid, precarious jobs during periods of illness and rehabilitation. While our findings focus on post-COVID-19 condition, similar deprivation-related patterns might exist for other post-acute infection syndromes or chronic disabilities. Implementing these measures could mitigate long-term social and economic consequences and prevent widening health inequalities, supporting a more equitable recovery for both current and future public health emergencies.^30^

Existing studies on functional impairments related to post-COVID-19 condition predominantly focused on hospitalised populations, with limited research in community-based cohorts. To our knowledge, this is the first study to investigate the associations between deprivation, migration status, ethnic minority status, and specific daily functional limitations from post-COVID-19 condition among community cases. Identifying individuals with post-COVID-19 condition using the standardised WHO definition, rather than clinical diagnoses, reduced bias from health-care-seeking behaviour and allowed for a more representative assessment of functional limitations. A detailed survey of functional limitations also allowed disaggregation across a range of daily activities, providing a more nuanced understanding of how this condition affects different population groups. These findings provided insights into the role of social determinants in shaping the functional impacts of post-COVID-19 condition and can inform targeted interventions and policy responses.

This study had several limitations. Functional limitations data were only collected from participants who reported any persistent symptoms, preventing comparison with participants without long-term symptoms or adjustment for pre-infection functional status, which might have strengthened the robustness of our findings. Additionally, the lack of pre-infection disability data limited adjustment for their potential confounding effect on the association between deprivation and functional limitations related to post-COVID-19 condition. Virus Watch participants were self-selected into the study and, therefore, were not fully representative of the English population. The cohort tended to be older, with approximately 29% of participants retired, which contributed to fewer responses for the outcome “attending or participating in work or education” and reduced the robustness of findings for this measure. Individuals from more deprived areas, migrants, and ethnic minorities were also under-represented. This under-representation was likely due to language barriers, limited internet access, and economic circumstances, which might affect the generalisability of our findings and reduce statistical power. The selection bias inherent in the Virus Watch cohort also extended to this retrospective analysis and was compounded by differential response rates to the persistent symptom surveys. The inability to stratify ethnicity and migration status into more granular groups limited the investigation of within-group heterogeneity, and the small sample size precluded analysis of functional limitation severity or intersectional effects. Further research with larger, more diverse samples is needed to address these issues. Furthermore, some individuals with post-COVID-19 condition might not have been identified due to limited community testing, recall bias through self-reporting of new persistent symptoms, and undetected asymptomatic infections. Under-ascertainment of cases might also have occurred after the end of free national COVID-19 testing, particularly among deprived groups less able to purchase testing kits. Further recall bias might have been introduced through the self-reporting of functional limitations. In addition, the use of IMD, an area-level measure of deprivation, might not accurately reflect individual socioeconomic position; future studies should use individual-level indicators, such as occupation and income. Although COVID-19 vaccination reduces the risk of severe acute illness, evidence for its protective effect against post-COVID-19 condition remains limited and mixed. While this study did not assess the impact of vaccination on the functional limitations of this condition, this could be explored in future research. Lastly, since we did not use the post COVID-19 functional status scale to assess functional limitations, our results are not directly comparable with studies that did.

In conclusion, the current study suggests elevated odds of experiencing functional limitations, particularly attending or participating in work or education, concentrating, self-care, and conducting necessary activities outside the household, among people living in more deprived areas compared with those in the least deprived areas. Our findings underscore the need to strengthen primary care capacity, to ensure equitable access to rehabilitation and support services, in addition to policies that ensure workplaces and educational institutions adapt to the needs of those affected by post-COVID-19 condition. Further investigation into more granular ethnicity and migration status groups, alongside the intersectionality of multiple social dimensions on long-term functional impacts on post-COVID-19 condition, is recommended to better inform targeted public health efforts, resource allocation, and policy responses for those most affected.

## Data sharing

We aim to share aggregate data and analysis code from this project on our website and via a "Findings so far" section on our website (https://ucl-virus-watch.net/) and via our GitHub repository (https://github.com/UCL-Public-Health-Data-Science). We will also be sharing individual record-level data on a research data sharing service such as the Office for National Statistics Secure Research Service. In sharing the data, we will work within the principles set out in the UK Research and Innovation Guidance on best practice in the management of research data. Access to use of the data while research is being conducted will be managed by the Chief Investigators (ACH, RWA, and IA). We will put analysis code on publicly available repositories to enable their reuse.

## Declaration of interests

We declare no competing interests.
