# Supplementary material for: Post-COVID-19 condition and disparities in daily functional activities in England: a retrospective analysis of data from the Virus Watch community cohort
Source: Lancet Prim Care. 2026 Jan;2(1):None. doi: 10.1016/j.lanprc.2025.100093 (PMC12867958; doi:10.1016/j.lanprc.2025.100093)
Supplement: Supplementary appendix [file mmc1.pdf]

THE LANCET
Primary Care

Supplementary appendix

This appendix formed part of the original submission and has been peer reviewed.
We post it as supplied by the authors.

Supplement to: Fong WLE, Beale S, Nguyen VG, et al. Post-COVID-19 condition and disparities in daily functional activities in England: a retrospective analysis of data from the Virus Watch community cohort. *Lancet Prim Care* 2026. https://doi.org/10.1016/j.lanprc.2025.100093

Appendix

Contents

STROBE checklist …………..………………..………..……………………………………………….. 3

Virus Watch Long-Term Symptoms Questionnaire: Symptom list……………………………………….. 5

Sources of SARS-CoV-2 Infection………..……….…………………………………………………….. 7

Directed Acyclic Graphs
Figure 1 Directed Acyclic Graph for the impact of deprivation (IMD Quintile) on experiencing functional limitations among people with Post-COVID-19 condition (PCC). This adjustment set holds the assumptions of deprivation influencing pre-infection health. 8
Figure 2 Directed Acyclic Graph for the impact of deprivation (IMD Quintile) on experiencing functional limitations among people with Post-COVID-19 condition (PCC). This adjustment set holds the assumptions of pre-infection health influencing deprivation. 9
Figure 3 Directed Acyclic Graph for the impact of migration status on experiencing functional limitations among people with Post-COVID-19 condition (PCC). This adjustment set holds the assumptions of migration status quintile influencing pre-infection health. 10
Figure 4 Directed Acyclic Graph for the impact of migration status on experiencing functional limitations among people with Post-COVID-19 condition (PCC). This adjustment set holds the assumptions of migration status influencing pre-infection health. 11
Figure 5 Directed Acyclic Graph for the impact of ethnicity on experiencing functional limitations among people with Post-COVID-19 condition (PCC). 12

Sociodemographic and Clinical Characteristics
Table 1 Sociodemographic and clinical characteristics of the analysis cohorts by IMD Quintile. 13
Table 2 Sociodemographic and clinical characteristics of the analysis cohorts by migration status....................13
Table 3 Sociodemographic and clinical characteristics of the analysis cohorts by ethnic minority status. 14
Table 4 Sociodemographic and clinical characteristics of the analysis cohorts by functional limitation. 15
Table 5 Number and proportion of participants who reported the presence or absence of limitations in each daily functional activity by sex. 17

Multicollinearity - Adjusted generalised standard error inflation factor (aGSIF)
Table 6 Total effect of IMD quintile on experiencing functional limitations. 18
Table 7 Total effect of migration status on experiencing functional limitations. 18
Table 8 Total effect of minority ethnicity status on experiencing functional limitations. 19

Main analysis: IMD quintile (including those with missing migration) as exposure
Table 9 Outcome: Attending or participating in work or education (n = 504). 20
Table 10 Outcome: Concentrating (n = 715). 21
Table 11 Outcome: Self-care (n = 685)....................23
Table 12 Outcome: Taking care of others in the household (n = 570). 24
Table 13 Outcome: Doing necessary activities outside the house (n = 712). 25
Table 14 Outcome: Doing enjoyable activities (n = 720). 27

Main analysis: Migration status as exposure (complete case analysis)
Table 15 Outcome: Attending or participating in work or education (n = 399). 29
Table 16 Outcome: Concentrating (n = 567). 30
Table 17 Outcome: Self-care (n = 542)....................31
Table 18 Outcome: Taking care of others in the household (n = 452). 32
Table 19 Outcome: Doing necessary activities outside the house (n = 566). 33
Table 20 Outcome: Doing enjoyable activities (n = 573). 34

Main analysis: Ethnic minority status as exposure (complete case analysis)
Table 21 Outcome: Attending or participating in work or education (n = 520). 36
Table 22 Outcome: Concentrating (n = 732). 37
Table 23 Outcome: Self-care (n = 702)....................38
Table 24 Outcome: Taking care of others in the household (n = 586). 39
Table 25 Outcome: Doing necessary activities in the house (n = 729)....................40

Table 26 Outcome: Doing enjoyable activities (n = 737). 41

Sensitivity analysis 1: IMD Quintile as exposure (complete case analysis)
Table 27 Sensitivity Analyses - Outcome: Attending or participating in work or education (n = 396). 42
Table 28 Sensitivity Analyses - Outcome: Concentrating (n = 563). 43
Table 29 Sensitivity Analyses - Outcome: Self-care (n = 538). 44
Table 30 Sensitivity Analyses - Outcome: Taking care of others in the household (n = 448). 45
Table 31 Sensitivity Analyses - Outcome: Doing necessary activities outside the house (n = 562). 47
Table 32 Sensitivity Analyses - Outcome: Doing enjoyable activities (n = 569). 48

Sensitivity analysis 2: IMD Quintile (without the health domain) as exposure
Table 33 Sensitivity Analyses - Outcome: Attending or participating in work or education (n = 516). 49
Table 34 Sensitivity Analyses - Outcome: Concentrating (n = 727). 51
Table 35 Sensitivity Analyses - Outcome: Self-care (n = 697). 52
Table 36 Sensitivity Analyses - Outcome: Taking care of others in the household (n = 581). 53
Table 37 Sensitivity Analyses - Outcome: Doing necessary activities outside the house (n = 724). 55
Table 38 Sensitivity Analyses - Outcome: Doing enjoyable activities (n = 732). 56

Sensitivity analysis 3: Migration status (including those with missing migration status) as exposure
Table 39 Sensitivity Analyses - Outcome: Attending or participating in work or education (n = 520). 58
Table 40 Sensitivity Analyses - Outcome: Concentrating (n = 732). 59
Table 41 Sensitivity Analyses - Outcome: Self-care (n = 702). 60
Table 42 Sensitivity Analyses - Outcome: Taking care of others in the household (n = 586). 61
Table 43 Sensitivity Analyses - Outcome: Doing necessary activities outside the house (n = 729). 62
Table 44 Sensitivity Analyses - Outcome: Doing enjoyable activities (n = 737). 63

Virus Watch Long-term Symptom Survey example 64

STROBE Statement

	Item No	Recommendation	Page No
**Title and abstract**	1	(*a*) Indicate the study's design with a commonly used term in the title or the abstract<br>(*b*) Provide in the abstract an informative and balanced summary of what was done and what was found	2
**Introduction**			
Background/rationale	2	Explain the scientific background and rationale for the investigation being reported	4
Objectives	3	State specific objectives, including any prespecified hypotheses	4
**Methods**			
Study design	4	Present key elements of study design early in the paper	4, 5
Setting	5	Describe the setting, locations, and relevant dates, including periods of recruitment, exposure, follow-up, and data collection	4, 5
Participants	6	(*a*) Give the eligibility criteria, and the sources and methods of selection of participants. Describe methods of follow-up<br>(*b*) For matched studies, give matching criteria and number of exposed and unexposed	5
Variables	7	Clearly define all outcomes, exposures, predictors, potential confounders, and effect modifiers. Give diagnostic criteria, if applicable	5, 6
Data sources/ measurement	8*	For each variable of interest, give sources of data and details of methods of assessment (measurement). Describe comparability of assessment methods if there is more than one group	5, 6
Bias	9	Describe any efforts to address potential sources of bias	5
Study size	10	Explain how the study size was arrived at	5
Quantitative variables	11	Explain how quantitative variables were handled in the analyses. If applicable, describe which groupings were chosen and why	5, 6
Statistical methods	12	(*a*) Describe all statistical methods, including those used to control for confounding<br>(*b*) Describe any methods used to examine subgroups and interactions<br>(*c*) Explain how missing data were addressed<br>(*d*) If applicable, explain how loss to follow-up was addressed<br>(*e*) Describe any sensitivity analyses	6
**Results**			
Participants	13*	(a) Report numbers of individuals at each stage of study—eg numbers potentially eligible, examined for eligibility, confirmed eligible, included in the study, completing follow-up, and analysed<br>(b) Give reasons for non-participation at each stage<br>(c) Consider use of a flow diagram	7, Fig 1
Descriptive data	14*	(a) Give characteristics of study participants (eg demographic, clinical, social) and information on exposures and potential confounders<br>(b) Indicate number of participants with missing data for each variable of interest<br>(c) Summarise follow-up time (eg, average and total amount)	7, 8
Outcome data	15*	Report numbers of outcome events or summary measures over time	7, Fig 1

Main results	16	(*a*) Give unadjusted estimates and, if applicable, confounder-adjusted estimates and their precision (eg, 95% confidence interval). Make clear which confounders were adjusted for and why they were included<br><br>(*b*) Report category boundaries when continuous variables were categorized<br>(*c*) If relevant, consider translating estimates of relative risk into absolute risk for a meaningful time period	8, 9, 10
Other analyses	17	Report other analyses done—eg analyses of subgroups and interactions, and sensitivity analyses	9
**Discussion**			
Key results	18	Summarise key results with reference to study objectives	11
Limitations	19	Discuss limitations of the study, taking into account sources of potential bias or imprecision. Discuss both direction and magnitude of any potential bias	12
Interpretation	20	Give a cautious overall interpretation of results considering objectives, limitations, multiplicity of analyses, results from similar studies, and other relevant evidence	11, 12
Generalisability	21	Discuss the generalisability (external validity) of the study results	12
**Other information**			
Funding	22	Give the source of funding and the role of the funders for the present study and, if applicable, for the original study on which the present article is based	12

*Give information separately for exposed and unexposed groups.

Note: An Explanation and Elaboration article discusses each checklist item and gives methodological background and published examples of transparent reporting. The STROBE checklist is best used in conjunction with this article (freely available on the Web sites of PLoS Medicine at http://www.plosmedicine.org/, Annals of Internal Medicine at http://www.annals.org/, and Epidemiology at http://www.epidem.com/). Information on the STROBE Initiative is available at http://www.strobe-statement.org.

Virus Watch Long-Term Symptoms Questionnaire: Symptom list

Note: There were two versions of the symptom list. The original symptom list was revised before the March 2023 survey to reflect evolving knowledge of post-COVID symptoms and to reduce survey burden by collapsing some items into single items. The 'Other' option was available in both questionnaires, and the majority of symptoms remained consistent or comparable. Symptom lists are provided here for reference.

Original Symptom List (February 2021 - March 2022)

Fatigue / Feeling unusually tired	Rash (all over)	Chills	Swollen glands
Headache	Fever	Night Sweats	Sinus pain / congestion
Feeling anxious	Feeling feverish	Rash (local)	Sneezing
Feeling depressed/low mood	Loss of Appetite	Vomiting (being sick)	Wheezing
Runny Nose; Blocked Nose	Trouble Sleeping	Nausea (feeling sick)	Chest tightness
Lightheaded or dizzy	Lack of concentration	Heart Palpitations (Fast or pounding heartbeat)	Sticky eye
Memory loss	Confusion, disorientation	Chest pain (not changed by breathing)	Eye pain
Dry cough;	Eye redness	Chest pain when breathing in	Ear pain
Wet cough	Deterioration of eyesight	Abdominal pain (not inc. menstrual pain)	Tinnitus (ringing or other noises in one or both ears)
Loss of sense of taste	Sore throat	Shortness of breath/difficulty breathing	Change in hearing
Altered/distorted sense of taste	Weakness	Muscle Aches	Other symptom(s)
Loss of sense of smell;	Weight loss	Bone or Joint Aches	
Reduced sense of smell	Altered/distorted sense of smell;	Other long-term pain	
Hair loss	Phantom smells in the absence of any odour	Swollen tonsils	

New Symptom List (March 2023 – April 2024)

Fatigue	Tachycardia (fast heartbeat) or palpitations	Unintentional weight gain
Headache	Chest pain	Problems with movement, balance, and/or coordination
Feeling anxious	Abdominal pain	Paraesthesia (pricking, tingling, or creeping feeling on the skin)
Feeling depressed	Shortness of breath/dyspnoea	Swelling of hands and/or feet
Congested/runny nose	Muscle aches	Difficulty swallowing (dysphagia)
Dizziness	Bone and joint pain	Easy bruising/ bleeding
Memory problems	Swollen lymph nodes	Acid reflux/heartburn
Cough	Fever	Gynaecological problems (e.g., change in menstrual cycles or flow)
Change/loss of taste	Change in Appetite	Urinary symptoms (e.g., bladder frequency, urgency or incontinence)
Change/loss of smell	Sleep problems (including difficulty falling asleep, staying asleep, oversleeping or sleep apnoea)	New allergy (e.g., to food, medications, etc.)
Hair loss	Brain fog and/or other cognitive problems	Ear pain
Skin rash	Dry eyes/ redness of eyes	Change in hearing
Unintentional weight loss	Visual changes; Blurry vision	Tinnitus
Nausea and/or vomiting	Sore throat or mouth	Other symptom(s), please describe:
Diarrhoea	Post-exertion malaise (feeling unwell or 'crashing' after exercise or physical exertion)	

Sources of SARS-CoV-2 Infection

We used the following sources to identify SARS-CoV-2 infections among study participants:

1. Polymerase chain reaction (PCR) or lateral flow test (LFT) results from linkage to the Second Generation Surveillance System (SGSS). Linkage was conducted by NHS Digital, and the linkage variables were sent in March 2021. Participant data were linked over time and between databases using the unique personal identifier recorded at all interactions with the NHS number, full name, date of birth and home address. The linkage period for SGSS Pillar 1 (secondary care testing) encompassed data from March 2020 until August 2021 and from June 2020 until August 2023 for SGSS Pillar 2 (community testing).

2. Self-reported SARS-CoV-2 test results (PCR or LFT) received from outside the study (e.g. via the UK Test Trace and Isolate system or privately obtained) as part of the weekly illness survey.

3. PCR test results from swabs provided by Virus Watch to a subset of participants between October 2020 and May 2021. Participants self-administered a PCR swab if they experienced fever, cough, loss or change of taste or smell/taste.

4. Anti-nucleocapsid or anti-spike antibody (prior to vaccination) serological test results provided by Virus Watch to a subset of participants. Participants attended in-clinic serological testing (September 2020-January 2021 and April 2021-July 2021) and/or conducted monthly at-home finger-prick serology (February 2021-April 2022). Anti-nucleocapsid or anti-spike antibodies presence in serology indicates prior infection. Infection date was estimated by identifying seroconversion during routine monthly testing. Further testing details are provided in the study protocol (1)

References

1. Hayward A, Fragaszy E, Kovar J, Nguyen V, Beale S, Byrne T, et al. Risk factors, symptom reporting, healthcare-seeking behaviour and adherence to public health guidance: protocol for Virus Watch, a prospective community cohort study. BMJ Open. 2021 Jun;11(6):e048042.

Directed Acyclic Graph

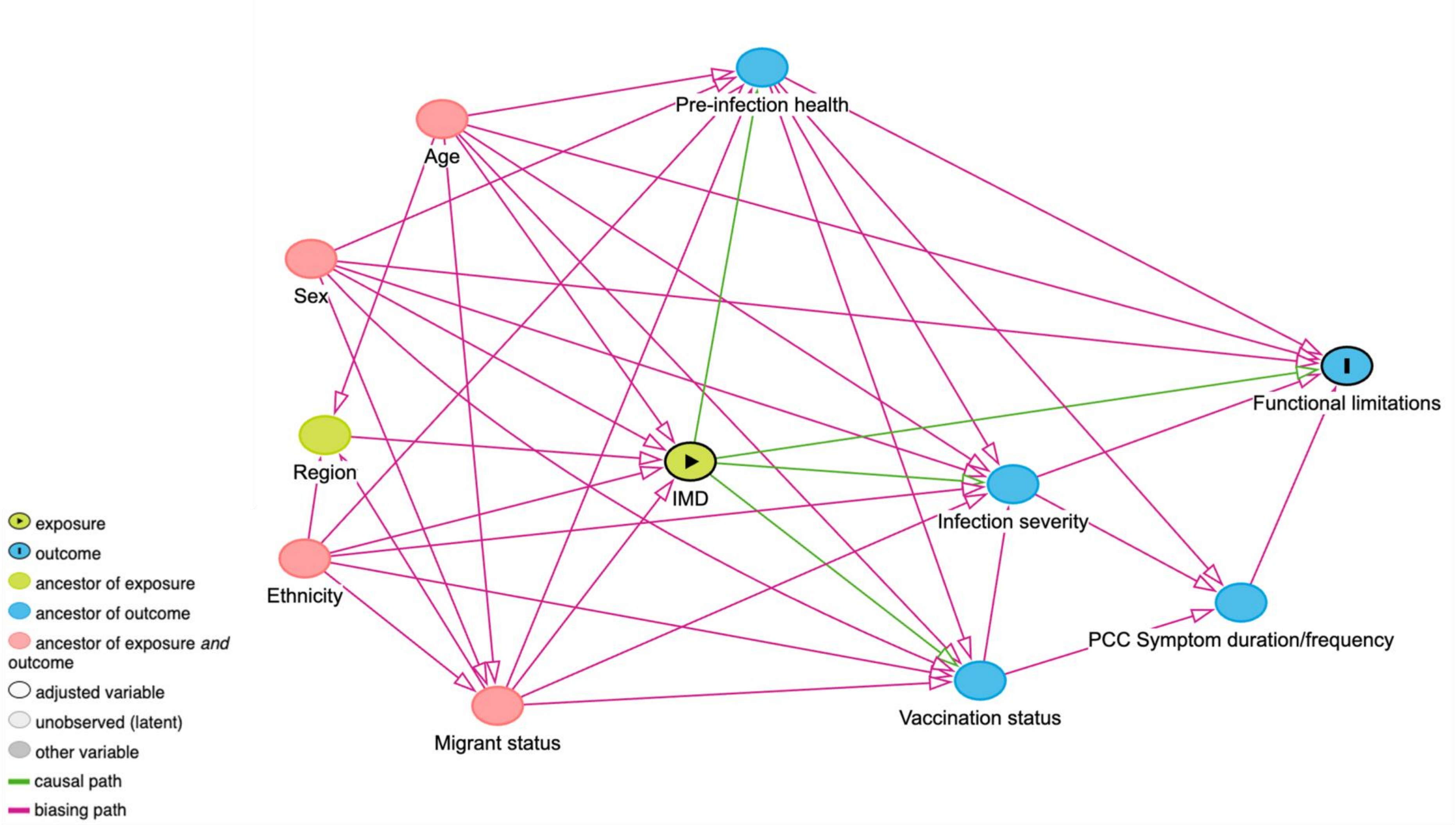

Figure 1 Directed Acyclic Graph for the impact of deprivation (IMD Quintile) on experiencing functional limitations among people with PCC. This adjustment set holds the assumptions of deprivation influencing pre-infection health.

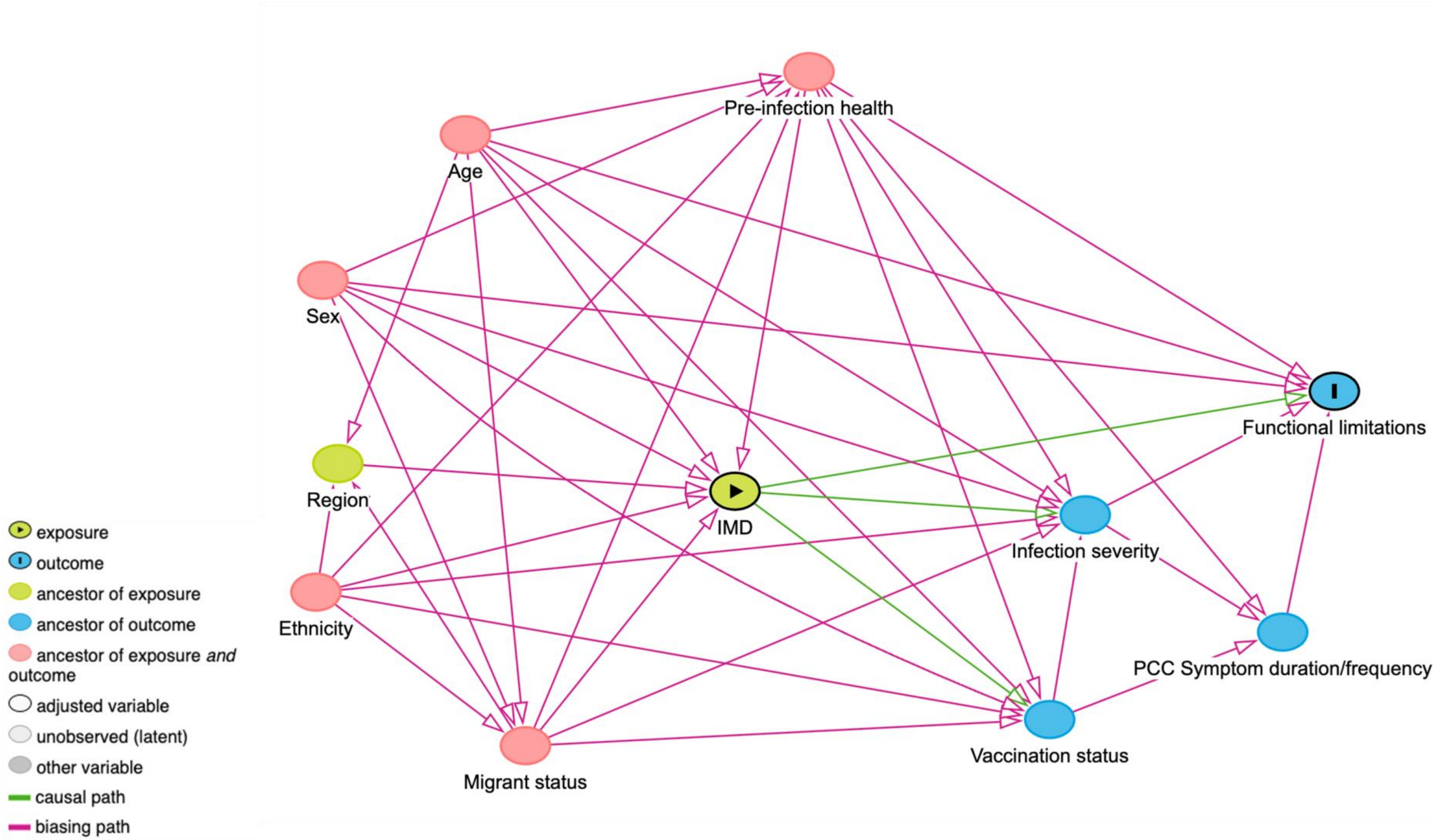

Figure 2 Directed Acyclic Graph for the impact of deprivation (IMD Quintile) on experiencing functional limitations among people with PCC. This adjustment set holds the assumptions of pre-infection health influencing deprivation.

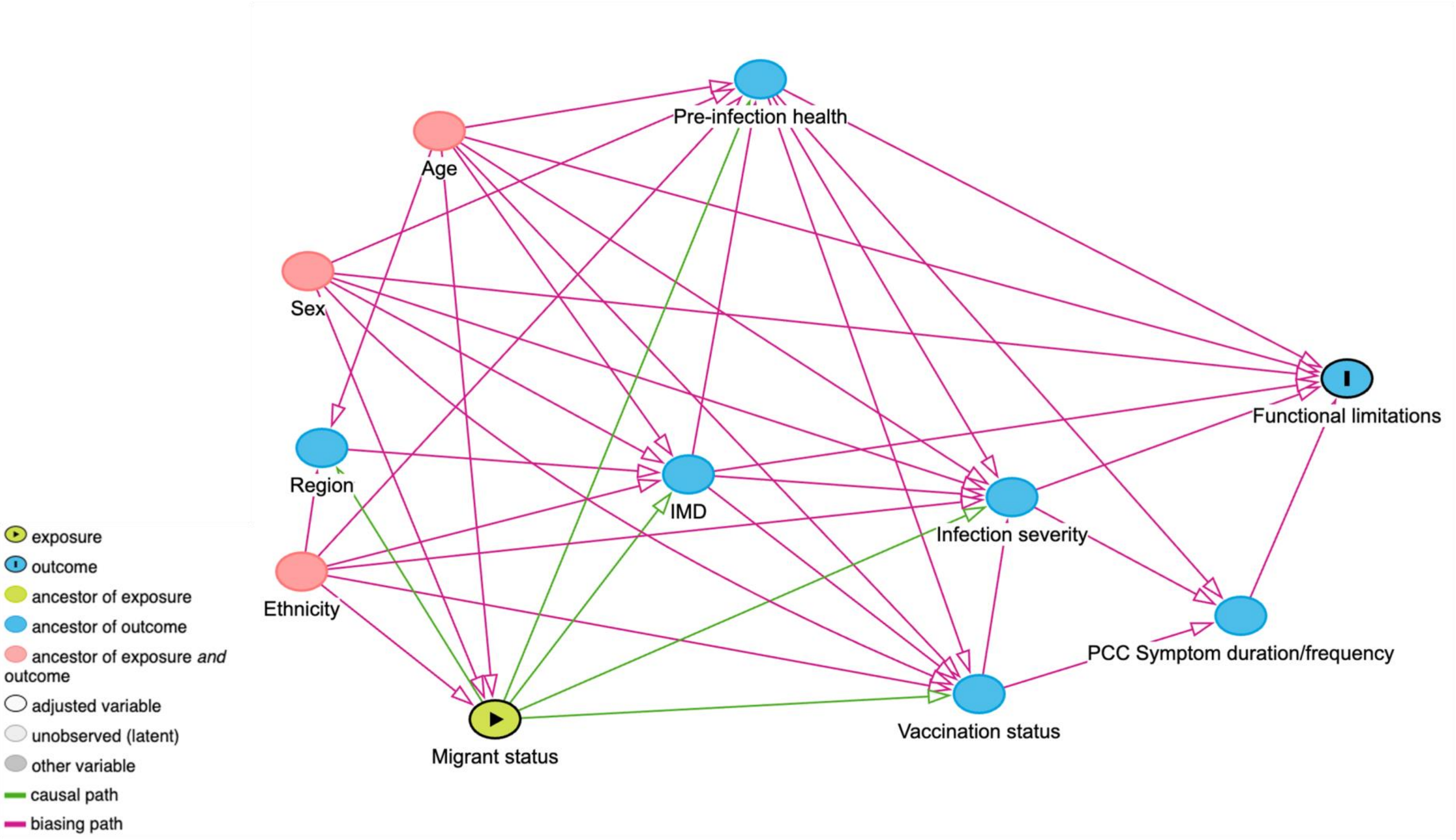

Figure 3 Directed Acyclic Graph for the impact of migration status on experiencing functional limitations among people with PCC. This adjustment set holds the assumptions of migration status quintile influencing pre-infection health.

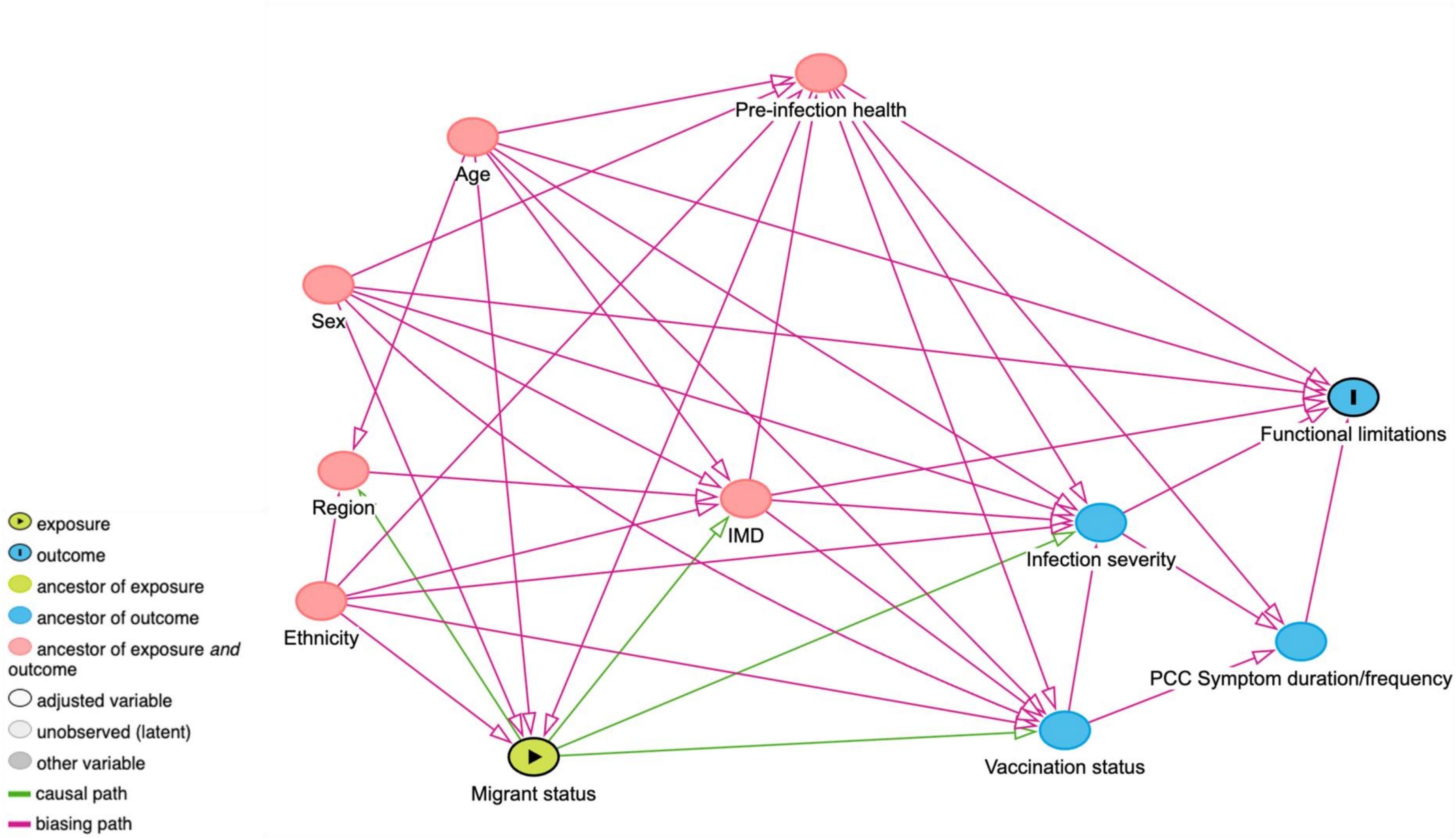

Figure 4 Directed Acyclic Graph for the impact of migration status on experiencing functional limitations among people with PCC. This adjustment set holds the assumptions of migration status influencing pre-infection health.

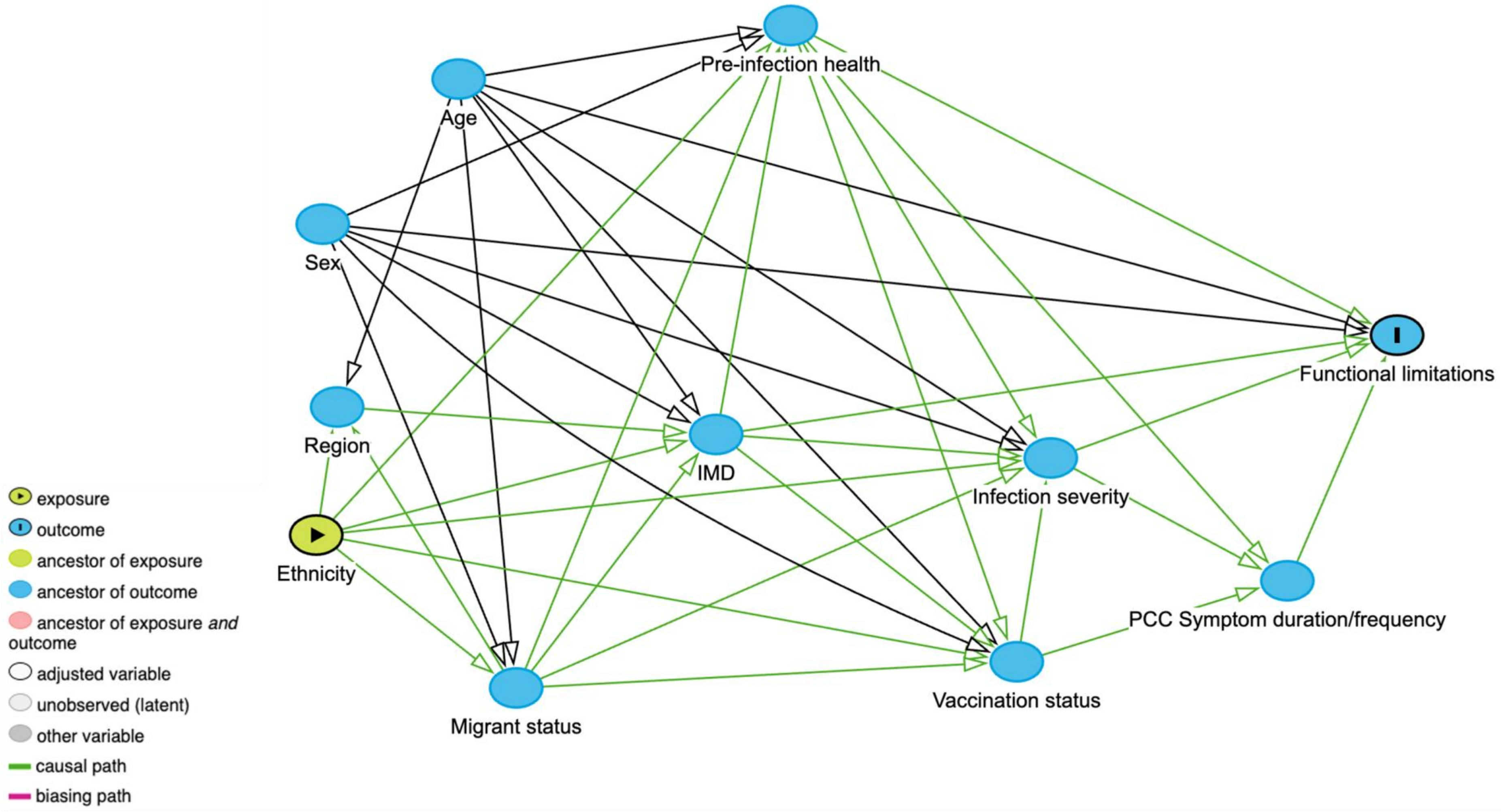

Figure 5 Directed Acyclic Graph for the impact of ethnicity on experiencing functional limitations among people with PCC.

Table 1 Sociodemographic and clinical characteristics of the analysis cohorts by IMD Quintile.

	IMD 1 (most deprived)	IMD 2	IMD 3	IMD 4	IMD 5 (least deprived)	Missing
**Characteristic**	**N = 68**	**N = 139**	**N = 165**	**N = 188**	**N = 207**	**N = 9**
**Age group**						
18-44	12 (17.6%)	25 (18.0%)	20 (12.1%)	14 (7.4%)	21 (10.1%)	*
45-64	37 (54.4%)	66 (47.5%)	86 (52.1%)	88 (46.8%)	95 (45.9%)	*
65+	19 (27.9%)	48 (34.5%)	59 (35.8%)	86 (45.7%)	91 (44.0%)	*
**Sex**						
Male	20 (29.4%)	39 (28.1%)	47 (28.5%)	47 (25.0%)	69 (33.3%)	*
Female	48 (70.6%)	100 (71.9%)	118 (71.5%)	141 (75.0%)	138 (66.7%)	*
**Migration status**						
UK Born	45 (66.2%)	98 (70.5%)	112 (67.9%)	138 (73.4%)	140 (67.6%)	*
Not UK Born/Missing*	23 (33.8%)	41 (29.5%)	53 (31.1%)	50 (26.6%)	67 (32.4%)	*
**Ethnic minority status**						
White British	57 (83.8%)	119 (85.6%)	142 (86.1%)	*	181 (87.4%)	*
Ethnic minority/Missing/Prefer not to say*	11 (16.2%)	20 (13.3%)	23 (13.9%)	*	26 (12.5%)	*
**Clinical vulnerability**						
Not clinically vulnerable/Missing*	32 (47.0%)	80 (57.6%)	83 (50.5%)	94 (50.0%)	103 (49.7%)	*
Clinically vulnerable	20 (29.4%)	36 (25.9%)	55 (33.3%)	65 (34.6%)	74 (35.7%)	*
Clinically extremely vulnerable	16 (23.5%)	23 (16.5%)	27 (16.4%)	29 (15.4%)	30 (14.5%)	*

* Cells with counts <10 have been combined with another category or suppressed to avoid disclosure.

Table 2 Sociodemographic and clinical characteristics of the analysis cohorts by migration status.

	UK Born	Not UK Born	Missing
**Characteristic**	**N = 538**	**N = 57**	**N = 181**
**Age group**			
18-44	56 (10.4%)	18 (31.6%)	19 (10.5%)
45-64	260 (48.3%)	29 (50.9%)	89 (49.2%)
65+	222 (41.3%)	10 (17.5%)	73 (40.3%)
**Sex**			
Male	162 (30.1%)	14 (24.6%)	48 (26.5%)
Female	376 (69.9%)	43 (75.4%)	133 (73.5%)

**IMD Quintile**			
1 (most deprived) & 2	143 (26.6%)	22 (38.6%)	42 (23.2%)
3	112 (20.8%)	18 (31.6%)	35 (19.3%)
4 & 5 (least deprived)/Missing*	283 (52.6%)	17 (29.9%)	104 (57.5%)
**IMD Quintile (excluding health domain)**			
1	43 (8.0%)	10 (17.5%)	13 (7.2%)
2	98 (18.2%)	17 (29.8%)	26 (14.4%)
3	124 (23.0%)	12 (21.1%)	47 (26.0%)
4 & 5 (least deprived)/Missing*	273 (50.7%)	18 (31.6%)	95 (52.4%)
**Ethnic minority status**			
White British	518 (96.3%)	13 (22.8%)	154 (85.1%)
Ethnic minority	20 (3.7%)	44 (77.2%)	16 (8.8%)
Missing/Prefer not to say	0 (0.0%)	0 (0.0%)	11 (6.1%)
**Clinical vulnerability**			
Not clinically vulnerable/Missing*	259 (48.1%)	25 (43.9%)	114 (63.0%)
Clinically vulnerable	179 (33.3%)	18 (31.6%)	54 (29.8%)
Clinically extremely vulnerable	100 (18.6%)	14 (24.6%)	13 (7.2%)

Table 3 Sociodemographic and clinical characteristics of the analysis cohorts by ethnic minority status.

	**White British**	**Ethnic minority**	**Missing/Prefer not to say**
**Characteristic**	**N = 685**	**N = 80**	**N = 11**
**Age group**			
18-44	62 (9.1%)	30 (37.5%)	*
45-64	335 (48.9%)	35 (43.8%)	*
65+	288 (42.0%)	15 (18.8%)	*
**Sex**			
Male	205 (29.9%)	16 (20.0%)	*
Female	480 (70.1%)	64 (80.0%)	*
**IMD Quintile**			
1 (most deprived)	57 (8.3%)	11 (13.8%)	*
2	119 (17.4%)	18 (22.5%)	*
3	142 (20.7%)	21 (26.3%)	*
4 & 5 (least deprived)/Missing	367 (53.5%)	30 (27.6%)	*
**IMD Quintile (excluding health domain)**			

1 (most deprived)	53 (7.7%)	13 (16.3%)	*
2	118 (17.2%)	21 (26.3%)	*
3	164 (23.9%)	17 (21.3%)	*
4 & 5 (least deprived)/Missing	350 (51.1%)	29 (36.3%)	*
**Migration status**			
UK Born	518 (75.6%)	20 (25.0%)	*
Not UK Born	13 (1.9%)	44 (55.0%)	*
Missing	154 (22.5%)	16 (20.0%)	*
**Clinical vulnerability**			
Not clinically vulnerable/Missing	350 (51.1%)	39 (48.8%)	*
Clinically vulnerable	220 (32.1%)	30 (37.5%)	*
Clinically extremely vulnerable	115 (16.8%)	11 (13.8%)	*

Table 4 Sociodemographic and clinical characteristics of the analysis cohorts by functional limitation.

	**Attend or participate in work or education**	**Concentrate on things**	**Take care of yourself**	**Take care of others in the household**	**Do necessary daily activities outside the household**	**Do activities that you enjoy**
**Characteristic**	**N = 530**	**N = 743**	**N = 713**	**N = 593**	**N = 740**	**N = 748**
**Age group**						
18-44	92 (17%)	91 (12%)	92 (13%)	80 (13%)	91 (12%)	91 (12%)
45-64	304 (57%)	368 (50%)	351 (49%)	300 (51%)	362 (49%)	364 (49%)
65+	134 (25%)	284 (38%)	270 (38%)	213 (36%)	287 (39%)	293 (39%)
**Sex**						
Male	152 (29%)	211 (28%)	206 (29%)	176 (30%)	212 (29%)	217 (29%)
Female	378 (71%)	532 (72%)	507 (71%)	417 (70%)	528 (71%)	531 (71%)
**IMD Quintile**						
1	52 (9.8%)	65 (8.7%)	59 (8.3%)	51 (8.6%)	66 (8.9%)	63 (8.4%)
2	107 (20%)	134 (18%)	129 (18%)	100 (17%)	131 (18%)	135 (18%)

3	116 (22%)	158 (21%)	154 (22%)	121 (20%)	158 (21%)	160 (21%)
4	122 (23%)	180 (24%)	175 (25%)	139 (23%)	183 (25%)	182 (24%)
5	125 (24%)	197 (27%)	187 (26%)	174 (29%)	193 (26%)	199 (27%)
Missing	8 (1.5%)	9 (1.2%)	9 (1.3%)	8 (1.3%)	9 (1.2%)	9 (1.2%)
**IMD Quintile (excluding the health domain)**						
1	50 (9.4%)	65 (8.7%)	59 (8.3%)	50 (8.4%)	64 (8.6%)	63 (8.4%)
2	108 (20%)	134 (18%)	129 (18%)	100 (17%)	132 (18%)	135 (18%)
3	129 (24%)	176 (24%)	171 (24%)	134 (23%)	177 (24%)	175 (23%)
4	119 (22%)	182 (24%)	179 (25%)	146 (25%)	186 (25%)	188 (25%)
5	116 (22%)	177 (24%)	166 (23%)	155 (26%)	172 (23%)	178 (24%)
Missing	8 (1.5%)	9 (1.2%)	9 (1.3%)	8 (1.3%)	9 (1.2%)	9 (1.2%)
**Migration status**						
UK Born	352 (66%)	514 (69%)	487 (68%)	409 (69%)	512 (69%)	518 (69%)
Not UK-born	48 (9.1%)	54 (7.3%)	56 (7.9%)	44 (7.4%)	55 (7.4%)	56 (7.5%)
Missing	130 (25%)	175 (24%)	170 (24%)	140 (24%)	173 (23%)	174 (23%)
**Ethnic minority status**						
White British	451 (85%)	655 (88%)	623 (87%)	522 (88%)	650 (88%)	658 (88%)
Ethnic minority	69 (13%)	77 (10%)	79 (11%)	64 (11%)	79 (11%)	79 (11%)
Missing/Prefer not to say	10 (1.9%)	11 (1.5%)	11 (1.5%)	7 (1.2%)	11 (1.5%)	11 (1.5%)
**Clinical vulnerability**						

Not clinically vulnerable	270 (51%)	359 (48%)	342 (48%)	292 (49%)	358 (48%)	361 (48%)
Clinically vulnerable	160 (30%)	243 (33%)	233 (33%)	186 (31%)	244 (33%)	243 (32%)
Clinically extremely vulnerable	82 (15%)	123 (17%)	120 (17%)	99 (17%)	120 (16%)	126 (17%)
Missing	18 (3.4%)	18 (2.4%)	18 (2.5%)	16 (2.7%)	18 (2.4%)	18 (2.4%)

Table 5 Number and proportion of participants who reported the presence or absence of limitations in each daily functional activity by sex.

	**Attend/participate in work/education**		**Concentrate on things**		**Self-care**		**Take care of others in the household**		**Do necessary daily activities outside the household**		**Do activities you enjoy**	
**Sex**	**Male (n = 152)**	**Female (n = 378)**	**Male (n = 211)**	**Female (n = 532)**	**Male (n = 206)**	**Female (n = 507)**	**Male (n = 176)**	**Female (n = 417)**	**Male (n = 212)**	**Female (n = 528)**	**Male (n = 217)**	**Female (n = 531)**
**Yes**	92 (60.5%)	233 (61.6%)	134 (73.5%)	361 (67.9%)	53 (25.7%)	133 (26.2%)	54 (30.7%)	144 (34.5%)	116 (54.7%)	332 (62.9%)	152 (70.0%)	393 (74.0%)
**No**	60 (39.5%)	145 (38.4%)	77 (36.5%)	171 (32.1%)	153 (74.3%)	374 (73.8%)	122 (69.3%)	273 (65.5%)	96 (45.3%)	196 (37.1%)	65 (30.0%)	138 (26.0%)

Multicollinearity

We produced generated the adjusted generalised standard error inflation factor (aGSIF) to detect multicollinearity in models. No evidence of multicollinearity was detected, with all aGSIF values below 2, respectively (see below Tables).

Table 6 Adjusted generalised standard error inflation factor (aGSIF) for regression models representing the total effect of IMD quintile on experiencing functional limitations. *aGSIF of below 2 suggest a lack of evidence of multicollinearity.*

**Exposure: IMD Quintile**	**aGSIF**					
**Analysis cohort**	Attending or participating in work/education	Concentrating	Self-care	Taking care of others in the household	Doing necessary activities outside the household	Doing enjoyable activities
IMD Quintile	1.01	1.01	1.01	1.01	1.01	1.01
Age group	1.02	1.02	1.03	1.02	1.02	1.02
Sex	1.01	1.01	1.01	1.01	1.01	1.01
Minority ethnicity status	1.26	1.27	1.33	1.27	1.31	1.29
Migration status	1.11	1.12	1.14	1.12	1.14	1.13

Table 7 Adjusted generalised standard error inflation factor (aGSIF) for regression models representing the total effect of migration status on experiencing functional limitations. *aGSIF of below 2 suggest a lack of evidence of multicollinearity.*

**Exposure: Migration Status**	**aGSIF**					
**Analysis cohort**	Attending or participating in work/education	Concentrating	Self-care	Taking care of others in the household	Doing necessary activities outside the household	Doing enjoyable activities
Migration status	1.33	1.37	1.41	1.37	1.40	1.37
Age group	1.01	1.01	1.02	1.01	1.02	1.02

Sex	1.00	1.00	1.01	1.00	1.01	1.01
Minority ethnicity status	1.34	1.38	1.43	1.38	1.42	1.39

Table 8 Adjusted generalised standard error inflation factor (aGSIF) for regression models representing the total effect of minority ethnicity status on experiencing functional limitations. *aGSIF of below 2 suggest a lack of evidence of multicollinearity.*

**Exposure: Ethnic Minority Status**	**aGSIF**					
**Analysis cohort**	Attending or participating in work/education	Concentrating	Self-care	Taking care of others in the household	Doing necessary activities outside the household	Doing enjoyable activities
Minority ethnicity status	1.04	1.03	1.04	1.03	1.04	1.04
Age group	1.02	1.02	1.02	1.01	1.02	1.02
Sex	1.00	1.00	1.01	1.00	1.00	1.01

Main Analysis – Odds Ratios (Exposure: IMD Quintile)

Table 9 Unadjusted odds ratio (OR) and adjusted odds ratio (aOR) for the association between IMD quintile and experiencing limitations in attending or participating in work or education (n = 504).

	**Had limitations**	**Did not have limitations**	Unadjusted			Adjusted			Adjusted + Clinical vulnerability		
	**n/N (%)**	**n/N (%)**	**OR**	**95% CI**	**p-value**	**aOR**	**95% CI**	**p-value**	**aOR**	**95% CI**	**p-value**
**IMD Quintile**											
1 (most deprived)	37/50 (74.0%)	13/50 (26.0%)	2.53	1.25, 5.39	0.012	2.30	1.12, 4.93	0.0264	2.31	1.13, 4.95	0.0262
2	70/102 (68.6%)	32/102 (31.4%)	1.95	1.13, 3.40	0.0174	1.90	1.09, 3.34	0.0246	1.96	1.12, 3.47	0.0188
3	66/112 (58.9%)	46/112 (41.1%)	1.28	0.76, 2.15	0.3543	1.23	0.73, 2.09	0.4406	1.22	0.72, 2.08	0.4649
4	69/119 (58%)	50/119 (42%)	1.23	0.74, 2.05	0.4278	1.26	0.75, 2.13	0.3873	1.27	0.75, 2.15	0.3768
5 (least deprived)	64/121 (52.9%)	57/121 (47.1%)	ref	-		ref	-	-	ref	-	-
**Age group**											
18-44	57/86 (66.3%)	29/86 (33.7%)				ref	-	-	ref	-	-
45-64	186/288 (64.6%)	102/288 (35.4%)				0.95	0.55, 1.6	0.8387	0.95	0.56, 1.60	0.8519
65+	63/130 (48.5%)	67/130 (51.5%)				0.52	0.29, 0.93	0.0298	0.52	0.29, 0.94	0.0304
**Sex**											
Male	88/144 (61.1%)	56/144 (38.9%)				ref	-	-	ref	-	-
Female	218/360 (60.6%)	142/360 (39.4%)				0.95	0.63, 1.43	0.8219	0.95	0.63, 1.43	0.8153
**Migration status**											
UK Born	211/348 (60.6%)	137/348 (39.4%)				ref	-	-	ref	-	-

Not UK Born	33/47 (70.2%)	14/47 (29.8%)				1.6	0.71, 3.77	0.2661	1.61	0.71, 3.80	0.2667
Missing	62/109 (56.9%)	47/109 (43.1%)				0.92	0.59, 1.45	0.7169	0.95	0.60, 1.49	0.8116
**Minority ethnicity status**											
White British	264/437 (60.4%)	173/437 (39.6%)				ref	-	-	ref	-	-
Ethnic minority	42/67 (62.7%)	25/67 (37.3%)				0.76	0.38, 1.52	0.4334	0.75	0.37, 1.50	0.4082
**Clinical vulnerability**											
Not clinically vulnerable	153/264 (58.0%)	111/264 (42.0%)							ref	-	-
Clinically vulnerable	103/160 (64.4%)	57/160 (35.6%)							1.37	0.90, 2.08	0.1446
Clinically extremely vulnerable	50/80 (62.5%)	30/80 (37.5%)							1.17	0.69, 2.01	0.5718
**Overall**	306/504 (60.7%)										

Table 10 Unadjusted odds ratio (OR) and adjusted odds ratio (aOR) for the association between IMD quintile and experiencing limitations in concentrating (n = 715).

	**Had limitations**	**Did not have limitations**	Unadjusted			Adjusted			Adjusted + Clinical vulnerability		
	**n/N (%)**	**n/N (%)**	**OR**	**95% CI**	**p-value**	**aOR**	**95% CI**	**p-value**	**aOR**	**95% CI**	**p-value**
**IMD Quintile**											
1 (most deprived)	51/63 (81.0%)	12/63 (19.0%)	2.97	1.53, 6.17	0.002	2.78	1.42, 5.81	0.004	2.76	1.41, 5.76	0.0045
2	95/129 (73.6%)	34/129 (26.4%)	1.95	1.21, 3.20	0.0069	1.91	1.17, 3.14	0.0098	1.92	1.18, 3.16	0.0092
3	105/154 (68.2%)	49/154 (31.8%)	1.50	0.96, 2.35	0.0747	1.46	0.93, 2.29	0.0997	1.46	0.93, 2.29	0.1004
4	110/177 (62.1%)	67/177 (37.9%)	1.15	0.76, 1.75	0.5182	1.16	0.76, 1.78	0.4948	1.16	0.76, 1.77	0.5019
5 (least deprived)	113/192 (58.9%)	79/192 (41.1%)	ref	-	-	ref	-	-	ref	-	-

**Age group**											
18-44	61/85 (71.8%)	24/85 (28.2%)				ref	-	-	ref	-	-
45-64	245/352 (69.6%)	107/352 (30.4%)				1.01	0.58, 1.73	0.9578	1.02	0.58, 1.74	0.948
65+	168/278 (60.4%)	110/278 (39.6%)				0.72	0.41, 1.25	0.2536	0.72	0.41, 1.24	0.2433
**Sex**											
Male	128/203 (63.1%)	75/203 (36.9%)				ref	-	-	ref	-	-
Female	346/512 (67.6%)	166/512 (32.4%)				1.16	0.82, 1.64	0.3962	1.17	0.82, 1.65	0.3790
**Migration status**											
UK Born	331/509 (65.0%)	178/509 (35.0%)				ref	-	-	ref	-	--
Not UK Born	38/53 (71.7%)	15/53 (28.3%)				0.89	0.4, 2	0.7682	0.87	0.39, 1.98	0.7409
Missing	105/153 (68.6%)	48/153 (31.4%)				1.19	0.8, 1.78	0.3893	1.21	0.82, 1.82	0.3435
**Minority ethnicity status**											
White British	418/640 (65.3%)	222/640 (34.7%)				ref	-	-	ref	-	-
Ethnic minority	56/75 (74.7%)	19/75 (25.3%)				1.44	0.72, 2.98	0.3127	1.44	0.73, 2.99	0.3091
**Clinical vulnerability**											
Not clinically vulnerable	230/353 (65.2%)	123/353 (34.8%)							ref	-	-
Clinically vulnerable	161/241 (66.8%)	80/241 (33.2%)							1.15	0.80, 1.64	0.4528
Clinically extremely vulnerable	83/121 (68.6%)	38/121 (31.4%)							1.2	0.76, 1.90	0.4383
**Overall**	474/715 (66.3%)										

Table 11 Unadjusted odds ratio (OR) and adjusted odds ratio (aOR) for the association between IMD quintile and experiencing limitations in self-care (n = 685).

	Had limitations	Did not have limitations	Unadjusted			Adjusted			Adjusted + Clinical vulnerability		
	**n/N (%)**	**n/N (%)**	**OR**	**95% CI**	**p-value**	**aOR**	**95% CI**	**p-value**	**aOR**	**95% CI**	**p-value**
**IMD Quintile**											
1 (most deprived)	19/57 (33.3%)	38/57 (66.7%)	2.26	1.15, 4.38	0.0168	2.11	1.06, 4.16	0.0311	2.04	1.02, 4.04	0.0408
2	34/124 (27.4%)	90/124 (72.6%)	1.71	0.99, 2.95	0.0552	1.64	0.95, 2.86	0.0774	1.68	0.96, 2.94	0.0671
3	41/150 (27.3%)	109/150 (72.7%)	1.70	1.01, 2.87	0.0462	1.70	1.00, 2.89	0.0496	1.67	0.99, 2.86	0.057
4	51/172 (29.7%)	121/172 (70.3%)	1.90	1.16, 3.16	0.0115	2.04	1.24, 3.42	0.0058	2.03	1.22, 3.40	0.0067
5 (least deprived)	33/182 (18.1%)	149/182 (81.9%)	ref	-	-	ref	-	-	ref	-	-
**Age group**											
18-44	33/86 (38.4%)	53/86 (61.6%)				ref	-	-	ref	-	-
45-64	79/335 (23.6%)	256/335 (76.4%)				0.52	0.31, 0.88	0.0143	0.52	0.31, 0.89	0.0168
65+	66/264 (25%)	198/264 (75%)				0.59	0.34, 1.02	0.0572	0.58	0.33, 1.01	0.0525
**Sex**											
Male	51/198 (25.8%)	147/198 (74.2%)				ref	-	-	ref	-	-
Female	127/487 (26.1%)	360/487 (73.9%)				0.93	0.63, 1.38	0.7134	0.95	0.65, 1.41	0.8028
**Migration status**											
UK Born	121/482 (25.1%)	361/482 (74.9%)				ref	-	-	ref	-	-
Not UK Born	19/55 (34.5%)	36/55 (65.5%)				1.29	0.58, 2.80	0.5249	1.22	0.54, 2.69	0.6222
Missing	38/148 (25.7%)	110/148 (74.3%)				1.04	0.67, 1.59	0.8497	1.12	0.72, 1.73	0.6043

**Minority ethnicity status**											
White British	152/608 (25%)	456/608 (75%)				ref	-	-	ref	-	-
Ethnic minority	26/77 (33.8%)	51/77 (66.2%)				1.2	0.60, 2.37	0.595	1.23	0.6, 2.45	0.559
**Clinical vulnerability**											
Not clinically vulnerable	71/336 (21.1%)	265/336 (78.9%)							ref	-	-
Clinically vulnerable	67/231 (29%)	164/231 (71%)							1.53	1.03, 2.26	0.0359
Clinically extremely vulnerable	40/118 (33.9%)	78/118 (66.1%)							1.91	1.18, 3.07	0.0078
**Overall**	178/685 (26%)										

Table 12 Unadjusted odds ratio (OR) and adjusted odds ratio (aOR) for the association between IMD quintile and experiencing limitations in taking care of others in the household (n = 570).

	**Had limitations**	**Did not have limitations**	Unadjusted			Adjusted			Adjusted + Clinical vulnerability		
	**n/N (%)**	**n/N (%)**	**OR**	**95% CI**	**p-value**	**aOR**	**95% CI**	**p-value**	**aOR**	**95% CI**	**p-value**
**IMD Quintile**											
1 (most deprived)	23/50 (46%)	27/50 (54%)	1.85	0.97, 3.51	0.0617	1.78	0.92, 3.41	0.0838	1.75	0.90, 3.37	0.096
2	33/96 (34.4%)	63/96 (65.6%)	1.13	0.66, 1.92	0.6400	1.09	0.63, 1.86	0.7563	1.11	0.64, 1.90	0.7056
3	34/117 (29.1%)	83/117 (70.9%)	0.89	0.53, 1.48	0.6486	0.86	0.51, 1.45	0.5840	0.86	0.51, 1.44	0.5628
4	48/136 (35.3%)	88/136 (64.7%)	1.18	0.73, 1.91	0.4926	1.14	0.70, 1.84	0.6063	1.14	0.70, 1.85	0.6073
5 (least deprived)	54/171 (31.6%)	117/171 (68.4%)	ref	-	-	ref	-	-	ref	-	-
**Age group**											
18-44	32/74 (43.2%)	42/74 (56.8%)				ref	-	-	ref	-	-

45-64	92/287 (32.1%)	195/287 (67.9%)				0.58	0.34, 1.01	0.0511	0.59	0.34, 1.01	0.0539
65+	68/209 (32.5%)	141/209 (67.5%)				0.60	0.34, 1.07	0.0823	0.6	0.34, 1.06	0.0762
**Sex**											
Male	51/168 (30.4%)	117/168 (69.6%)				ref	-	-	ref	-	-
Female	141/402 (35.1%)	261/402 (64.9%)				1.23	0.84, 1.84	0.2948	1.26	0.85, 1.89	0.2477
**Migration status**											
UK Born	140/404 (34.7%)	264/404 (65.3%)				ref	-	-	ref	-	-
Not UK Born	12/43 (27.9%)	31/43 (72.1%)				0.80	0.32, 1.91	0.6197	0.76	0.3, 1.84	0.557
Missing	40/123 (32.5%)	83/123 (67.5%)				0.93	0.60, 1.43	0.7318	0.97	0.62, 1.5	0.8811
**Minority ethnicity status**											
White British	174/508 (34.3%)	334/508 (65.7%)				ref	-	-	ref	-	-
Ethnic minority	18/62 (29%)	44/62 (71%)				0.78	0.36, 1.62	0.5175	0.8	0.37, 1.66	0.5495
**Clinical vulnerability**											
Not clinically vulnerable	85/287 (29.6%)	202/287 (70.4%)							ref	-	-
Clinically vulnerable	69/185 (37.3%)	116/185 (62.7%)							1.45	0.98, 2.16	0.065
Clinically extremely vulnerable	38/98 (38.8%)	60/98 (61.2%)							1.50	0.91, 2.44	0.1081
**Overall**	192/570 (33.7%)										

Table 13 Unadjusted odds ratio (OR) and adjusted odds ratio (aOR) for the association between IMD quintile and experiencing limitations in doing necessary activities outside the house (n = 712).

	**Had limitations**	**Did not have limitations**	Unadjusted			Adjusted			Adjusted + Clinical vulnerability		

	n/N (%)	n/N (%)	OR	95% CI	p-value	aOR	95% CI	p-value	aOR	95% CI	p-value
**IMD Quintile**											
1 (most deprived)	46/64 (71.9%)	18/64 (28.1%)	2.06	1.13, 3.90	0.0211	2.06	1.12, 3.90	0.0231	2.04	1.10, 3.91	0.0262
2	79/126 (62.7%)	47/126 (37.3%)	1.36	0.86, 2.16	0.1942	1.35	0.85, 2.15	0.2077	1.42	0.89, 2.28	0.1483
3	89/154 (57.8%)	65/154 (42.2%)	1.11	0.72, 1.70	0.6463	1.08	0.70, 1.67	0.7133	1.09	0.70, 1.69	0.6943
4	115/180 (63.9%)	65/180 (36.1%)	1.43	0.94, 2.18	0.0946	1.38	0.90, 2.11	0.1392	1.39	0.90, 2.13	0.1343
5 (least deprived)	104/188 (55.3%)	84/188 (44.7%)	ref	-		ref	-	-	ref	-	-
**Age group**											
18-44	54/85 (63.5%)	31/85 (36.5%)				ref	-	-	ref	-	-
45-64	206/346 (59.5%)	140/346 (40.5%)				0.82	0.49, 1.36	0.4544	0.84	0.50, 1.40	0.5089
65+	173/281 (61.6%)	108/281 (38.4%)				0.93	0.54, 1.57	0.7879	0.91	0.53, 1.54	0.7288
**Sex**											
Male	113/204 (55.4%)	91/204 (44.6%)				ref	-	-	ref	-	-
Female	320/508 (63%)	188/508 (37%)				1.38	0.98, 1.92	0.0621	1.41	1.01, 1.98	0.045
**Migration status**											
UK Born	309/507 (60.9%)	198/507 (39.1%)				ref	-	-	ref	-	-
Not UK Born	33/54 (61.1%)	21/54 (38.9%)				1.27	0.61, 2.73	0.5288	1.21	0.57, 2.62	0.6224
Missing	91/151 (60.3%)	60/151 (39.7%)				0.99	0.68, 1.45	0.9523	1.06	0.73, 1.57	0.7551
**Minority ethnicity status**											
White British	389/635 (61.3%)	246/635 (38.7%)				ref	-	-	ref	-	-
Ethnic minority	44/77 (57.1%)	33/77 (42.9%)				0.7	0.37, 1.33	0.271	0.7	0.37, 1.34	0.2796

**Clinical vulnerability**											
Not clinically vulnerable	189/352 (53.7%)	163/352 (46.3%)							ref	-	-
Clinically vulnerable	161/242 (66.5%)	81/242 (33.5%)							1.79	1.27, 2.53	0.001
Clinically extremely vulnerable	83/118 (70.3%)	35/118 (29.7%)							2.02	1.29, 3.22	0.0025
**Overall**	433/712 (60.8%)										

Table 14 Unadjusted odds ratio (OR) and adjusted odds ratio (aOR) for the association between IMD quintile and experiencing limitations in doing enjoyable activities (n = 720).

	**Had limitations**	**Did not have limitations**	Unadjusted			Adjusted			Adjusted + Clinical vulnerability		
	**n/N (%)**	**n/N (%)**	**OR**	**95% CI**	**p-value**	**aOR**	**95% CI**	**p-value**	**aOR**	**95% CI**	**p-value**
**IMD Quintile**											
1 (most deprived)	49/61 (80.3%)	12/61 (19.7%)	1.78	0.91, 3.74	0.1056	1.77	0.90, 3.73	0.1124	1.78	0.90, 3.74	0.1128
2	93/130 (71.5%)	37/130 (28.5%)	1.1	0.68, 1.80	0.7063	1.09	0.67, 1.79	0.732	1.11	0.68, 1.83	0.6665
3	111/156 (71.2%)	45/156 (28.8%)	1.08	0.68, 1.72	0.75	1.07	0.67, 1.71	0.7742	1.07	0.67, 1.71	0.7815
4	136/179 (76%)	43/179 (24%)	1.38	0.87, 2.2	0.1673	1.37	0.86, 2.18	0.1872	1.36	0.86, 2.18	0.191
5 (least deprived)	135/194 (69.6%)	59/194 (30.4%)	ref	-	-	ref	-	-	ref	-	-
**Age group**											
18-44	64/85 (75.3%)	21/85 (24.7%)				ref	-	-	ref	-	-
45-64	249/348 (71.6%)	99/348 (28.4%)				0.81	0.45, 1.40	0.4627	0.82	0.46, 1.42	0.4852
65+	211/287 (73.5%)	76/287 (26.5%)				0.92	0.50, 1.62	0.769	0.91	0.50, 1.61	0.7447
**Sex**											

Male	147/209 (70.3%)	62/209 (29.7%)				ref	-	-	ref	-	-
Female	377/511 (73.8%)	134/511 (26.2%)				1.18	0.82, 1.69	0.371	1.19	0.83, 1.71	0.3374
**Migration status**											
UK Born	372/513 (72.5%)	141/513 (27.5%)				ref	-	-	ref	-	-
Not UK Born	41/55 (74.5%)	14/55 (25.5%)				1.32	0.59, 3.04	0.504	1.31	0.59, 3.02	0.5222
Missing	111/152 (73%)	41/152 (27%)				1.04	0.69, 1.58	0.8573	1.08	0.71, 1.64	0.7339
**Minority ethnicity status**											
White British	469/643 (72.9%)	174/643 (27.1%)				ref	-	-	ref	-	-
Ethnic minority	55/77 (71.4%)	22/77 (28.6%)				0.78	0.40, 1.57	0.476	0.77	0.39, 1.56	0.4642
**Clinical vulnerability**											
Not clinically vulnerable	248/355 (69.9%)	107/355 (30.1%)							ref	-	-
Clinically vulnerable	183/241 (75.9%)	58/241 (24.1%)							1.37	0.95, 2.01	0.0984
Clinically extremely vulnerable	93/124 (75%)	31/124 (25%)							1.27	0.80, 2.06	0.3257
**Overall**	524/720 (72.8%)										

Main Analysis – Odds Ratios (Exposure: Migration Status)

Table 15 Unadjusted odds ratio (OR) and adjusted odds ratio (aOR) for the association between migration status and experiencing limitations in attending or participating in work or education (n = 399).

	Had limitations	Did not have limitations	Unadjusted			Adjusted			Adjusted + Clinical vulnerability		
	n/N (%)	n/N (%)	OR	95% CI	p-value	aOR	95% CI	p-value	aOR	95% CI	p-value
**Migration Status**											
UK Born	215/352 (61.1%)	137/352 (38.9%)	ref	-	-	ref	-	-	ref	-	-
Not UK Born	33/47 (70.2%)	14/47 (29.8%)	1.50	0.79, 2.99	0.2276	1.52	0.64, 3.77	0.3553	1.49	0.62, 3.73	0.3749
**Age group**											
18-44	49/72 (68.1%)	23/72 (31.9%)				ref	-	-	ref	-	-
45-64	155/234 (66.2%)	79/234 (33.8%)				0.94	0.52, 1.65	0.8283	0.94	0.52, 1.65	0.8288
65+	44/93 (47.3%)	49/93 (52.7%)				0.44	0.22, 0.83	0.0132	0.43	0.22, 0.83	0.0122
**Sex**											
Male	73/119 (61.3%)	46/119 (38.7%)				ref	-	-	ref	-	-
Female	175/280 (62.5%)	105/280 (37.5%)				1.01	0.64, 1.58	0.9723	1.01	0.64, 1.58	0.9595
**Minority Ethnicity Status**											
White British	212/344 (61.6%)	132/344 (38.4%)				ref	-	-	ref	-	-
Ethnic minority	36/55 (65.5%)	19/55 (34.5%)				0.81	0.36, 1.82	0.6017	0.81	0.36, 1.83	0.6007
**Clinical vulnerability**											
Not clinically vulnerable	120/199 (60.3%)	79/199 (39.7%)							ref	-	-
Clinically vulnerable	83/129 (64.3%)	46/129 (35.7%)							1.20	0.76, 1.92	0.4404
Clinically extremely vulnerable	45/71 (63.4%)	26/71 (36.6%)							1.20	0.68, 2.15	0.5366

**Overall**	248/399 (62.2%)										

Table 16 Unadjusted odds ratio (OR) and adjusted odds ratio (aOR) for the association between migration status and experiencing limitations in concentrating (n = 567).

	**Had limitations**	**Did not have limitations**	Unadjusted			Adjusted			Adjusted + Clinical vulnerability		
	**n/N (%)**	**n/N (%)**	**OR**	**95% CI**	**p-value**	**aOR**	**95% CI**	**p-value**	**aOR**	**95% CI**	**p-value**
**Migration Status**											
UK Born	335/514 (65.2%)	179/514 (34.8%)	ref	-	-	ref	-	-	ref	-	-
Not UK Born	38/53 (71.7%)	15/53 (28.3%)	1.35	0.74, 2.60	0.342	0.68	0.28, 1.64	0.3893	0.66	0.27, 1.59	0.3574
**Age group**											
18-44	50/71 (70.4%)	21/71 (29.6%)				ref	-	-	ref	-	-
45-64	197/281 (70.1%)	84/281 (29.9%)				1.08	0.60, 1.91	0.7934	1.08	0.60, 1.91	0.794
65+	126/215 (58.6%)	89/215 (41.4%)				0.68	0.37, 1.21	0.1948	0.67	0.37, 1.21	0.189
**Sex**											
Male	101/164 (61.6%)	63/164 (38.4%)				ref	-	-	ref	-	-
Female	272/403 (67.5%)	131/403 (32.5%)				1.22	0.83, 1.79	0.3034	1.22	0.83, 1.79	0.3041
**Minority Ethnicity Status**											
White British	326/507 (64.3%)	181/507 (35.7%)				ref	-	-	ref	-	-
Ethnic minority	47/60 (78.3%)	13/60 (21.7%)				2.34	1.00, 5.96	0.06	2.38	1.02, 6.07	0.0554
**Clinical vulnerability**											
Not clinically vulnerable	176/268 (65.7%)	92/268 (34.3%)							ref	-	-
Clinically vulnerable	122/189 (64.6%)	67/189 (35.4%)							0.99	0.67, 1.48	0.9672

Clinically extremely vulnerable	75/110 (68.2%)	35/110 (31.8%)							1.19	0.74, 1.94	0.4748
**Overall**	373/567 (65.8%)										

Table 17 Unadjusted odds ratio (OR) and adjusted odds ratio (aOR) for the association between migration status and experiencing limitations in self-care (n = 542).

	**Had limitations**	**Did not have limitations**	Unadjusted			Adjusted			Adjusted + Clinical vulnerability		
	**n/N (%)**	**n/N (%)**	**OR**	**95% CI**	**p-value**	**aOR**	**95% CI**	**p-value**	**aOR**	**95% CI**	**p-value**
**Migration Status**											
UK Born	123/487 (25.3%)	364/487 (74.7%)	ref	-	-	ref	-	-	ref	-	-
Not UK Born	19/55 (34.5%)	36/55 (65.5%)	1.56	0.85, 2.79	0.14	1.13	0.48, 2.60	0.7772	1.03	0.43, 2.41	0.9422
**Age group**											
18-44	29/72 (40.3%)	43/72 (59.7%)				ref	-	-	ref	-	-
45-64	65/267 (24.3%)	202/267 (75.7%)				0.5	0.29, 0.88	0.0155	0.5	0.29, 0.88	0.0152
65+	48/203 (23.6%)	155/203 (76.4%)				0.49	0.27, 0.89	0.0183	0.48	0.26, 0.87	0.0144
**Sex**											
Male	44/161 (27.3%)	117/161 (72.7%)				ref	-	-	ref	-	-
Female	98/381 (25.7%)	283/381 (74.3%)				0.87	0.58, 1.34	0.532	0.89	0.58, 1.37	0.5889
**Minority Ethnicity Status**											
White British	120/480 (25%)	360/480 (75%)				ref	-	-	ref	-	-
Ethnic minority	22/62 (35.5%)	40/62 (64.5%)				1.32	0.58, 2.93	0.4965	1.40	0.61, 3.14	0.4133
**Clinical vulnerability**											
Not clinically vulnerable	59/253 (23.3%)	194/253 (76.7%)							ref	-	-
Clinically vulnerable	46/182 (25.3%)	136/182 (74.7%)							1.10	0.70, 1.73	0.6745

Clinically extremely vulnerable	37/107 (34.6%)	70/107 (65.4%)							1.77	1.07, 2.92	0.0259
**Overall**	142/542 (26.2%)										

Table 18 Unadjusted odds ratio (OR) and adjusted odds ratio (aOR) for the association between migration status and experiencing limitations in taking care of others in the household (n = 452).

	**Had limitations**	**Did not have limitations**	Unadjusted			Adjusted			Adjusted + Clinical vulnerability		
	**n/N (%)**	**n/N (%)**	**OR**	**95% CI**	**p-value**	**aOR**	**95% CI**	**p-value**	**aOR**	**95% CI**	**p-value**
**Migration Status**											
UK Born	142/409 (34.7%)	267/409 (65.3%)	ref	-	-	ref	-	-	ref	-	-
Not UK Born	12/43 (27.9%)	31/43 (72.1%)	0.73	0.35, 1.43	0.3716	0.67	0.25, 1.73	0.4183	0.65	0.24, 1.68	0.3817
**Age group**											
18-44	28/63 (44.4%)	35/63 (55.6%)				ref	-	-	ref	-	-
45-64	73/227 (32.2%)	154/227 (67.8%)				0.57	0.32, 1.01	0.0535	0.56	0.32, 1.01	0.053
65+	53/162 (32.7%)	109/162 (67.3%)				0.57	0.31, 1.06	0.0752	0.56	0.30, 1.04	0.0665
**Sex**											
Male	42/137 (30.7%)	95/137 (69.3%)				ref	-	-	ref	-	-
Female	112/315 (35.6%)	203/315 (64.4%)				1.24	0.81, 1.92	0.3352	1.27	0.83, 1.98	0.2784
**Minority Ethnicity Status**											
White British	139/403 (34.5%)	264/403 (65.5%)				ref	-	-	ref	-	-
Ethnic minority	15/49 (30.6%)	34/49 (69.4%)				0.96	0.39, 2.30	0.9214	0.97	0.39, 2.35	0.9464
**Clinical vulnerability**											
Not clinically vulnerable	68/216 (31.5%)	148/216 (68.5%)							ref	-	-

Clinically vulnerable	54/148 (36.5%)	94/148 (63.5%)							1.30	0.83, 2.03	0.2551
Clinically extremely vulnerable	32/88 (36.4%)	56/88 (63.6%)							1.31	0.77, 2.21	0.3183
**Overall**	154/452 (34.1%)										

Table 19 Unadjusted odds ratio (OR) and adjusted odds ratio (aOR) for the association between migration status and experiencing limitations in doing necessary activities outside the house (n = 566).

	**Had limitations**	**Did not have limitations**	Unadjusted			Adjusted			Adjusted + Clinical vulnerability		
	**n/N (%)**	**n/N (%)**	**OR**	**95% CI**	**p-value**	**aOR**	**95% CI**	**p-value**	**aOR**	**95% CI**	**p-value**
**Migration Status**											
UK Born	313/512 (61.1%)	199/512 (38.9%)	ref	-	-	ref	-	-	ref	-	-
Not UK Born	33/54 (61.1%)	21/54 (38.9%)	1.00	0.57, 1.80	0.9975	1.24	0.56, 2.84	0.5964	1.16	0.51, 2.66	0.7293
**Age group**											
18-44	45/71 (63.4%)	26/71 (36.6%)				ref	-	-	ref	-	-
45-64	165/276 (59.8%)	111/276 (40.2%)				0.84	0.48, 1.45	0.5435	0.86	0.49, 1.48	0.5811
65+	136/219 (62.1%)	83/219 (37.9%)				0.95	0.53, 1.67	0.8508	0.91	0.51, 1.61	0.7495
**Sex**											
Male	91/166 (54.8%)	75/166 (45.2%)				ref	-	-	ref	-	-
Female	255/400 (63.7%)	145/400 (36.2%)				1.47	1.02, 2.13	0.0409	1.53	1.05, 2.23	0.026
**Minority Ethnicity Status**											
White British	310/504 (61.5%)	194/504 (38.5%)				ref	-	-	ref	-	-
Ethnic minority	36/62 (58.1%)	26/62 (41.9%)				0.72	0.33, 1.54	0.3884	0.73	0.34, 1.57	0.4158
**Clinical vulnerability**											

Not clinically vulnerable	146/268 (54.5%)	122/268 (45.5%)							ref	-	-
Clinically vulnerable	123/191 (64.4%)	68/191 (35.6%)							1.57	1.07, 2.32	0.0222
Clinically extremely vulnerable	77/107 (72%)	30/107 (28%)							2.17	1.34, 3.59	0.0019
**Overall**	346/566 (61.1%)										

Table 20 Unadjusted odds ratio (OR) and adjusted odds ratio (aOR) for the association between migration status and experiencing limitations in doing enjoyable activities (n = 573).

	**Had limitations**	**Did not have limitations**	Unadjusted			Adjusted			Adjusted + Clinical vulnerability		
	**n/N (%)**	**n/N (%)**	**OR**	**95% CI**	**p-value**	**aOR**	**95% CI**	**p-value**	**aOR**	**95% CI**	**p-value**
**Migration Status**											
UK Born	376/518 (72.6%)	142/518 (27.4%)	ref	-	-	ref	-	-	ref	-	-
Not UK Born	41/55 (74.5%)	14/55 (25.5%)	1.11	0.60, 2.16	0.7564	1.24	0.53, 3.05	0.6247	1.21	0.51, 2.97	0.6715
**Age group**											
18-44	53/71 (74.6%)	18/71 (25.4%)				ref	-	-	ref	-	-
45-64	197/278 (70.9%)	81/278 (29.1%)				0.83	0.44, 1.49	0.5392	0.83	0.44, 1.49	0.5426
65+	167/224 (74.6%)	57/224 (25.4%)				1.01	0.52, 1.87	0.9865	0.99	0.52, 1.85	0.9849
**Sex**											
Male	124/172 (72.1%)	48/172 (27.9%)				ref	-	-	ref	-	-
Female	293/401 (73.1%)	108/401 (26.9%)				1.06	0.71, 1.59	0.7612	1.07	0.71, 1.6	0.7355
**Minority Ethnicity Status**											
White British	372/511 (72.8%)	139/511 (27.2%)				ref	-	-	ref	-	-
Ethnic minority	45/62 (72.6%)	17/62 (27.4%)				0.86	0.38, 1.99	0.7147	0.87	0.39, 2.02	0.7444

**Clinical vulnerability**											
Not clinically vulnerable	190/267 (71.2%)	77/267 (28.8%)							ref	-	-
Clinically vulnerable	141/193 (73.1%)	52/193 (26.9%)							1.09	0.72, 1.66	0.6801
Clinically extremely vulnerable	86/113 (76.1%)	27/113 (23.9%)							1.27	0.77, 2.14	0.3603
**Overall**	417/573 (72.8%)										

Main Analyses – Odds Ratios (Exposure: Ethnic minority status)

Table 21 Unadjusted odds ratio (OR) and adjusted odds ratio (aOR) for the association between ethnic minority status and experiencing limitations in attending or participating in work or education (n = 520).

	Experienced limitations	Did not experience limitations	Unadjusted model			Adjusted model		
	n/N (%)	n/N (%)	OR	95% CI	p-value	aOR	95% CI	p-value
**Minority ethnicity status**								
White British	274/451 (60.8%)	177/451 (39.2%)	ref	-	-	ref	-	-
Ethnic minority	44/69 (63.8%)	25/69 (36.2%)	1.14	0.68, 1.95	0.6325	1.00	0.58, 1.76	0.9882
**Age group**								
18-44	61/91 (67.0%)	30/91 (33.0%)				ref	-	-
45-64	192/296 (64.9%)	104/296 (35.1%)				0.91	0.54, 1.51	0.7144
65+	65/133 (48.9%)	68/133 (51.1%)				0.47	0.26, 0.83	0.0096
**Sex**								
Male	91/149 (61.1%)	58/149 (38.9%)				ref	-	-
Female	227/371 (61.2%)	144/371 (38.8%)				1.00	0.67, 1.48	0.9964
**Overall**	318/520 (61.2%)							

Table 22 Unadjusted odds ratio (OR) and adjusted odds ratio (aOR) for the association between ethnic minority status and experiencing limitations in concentrating (n = 732).

	Experienced limitations	Did not experience limitations	Unadjusted model			Adjusted model		
	n/N (%)	n/N (%)	OR	95% CI	p-value	aOR	95% CI	p-value
**Minority ethnicity status**								
White British	430/655 (65.6%)	225/655 (34.4%)	ref	-	-	ref	-	-
Ethnic minority	58/77 (75.3%)	19/77 (24.7%)	1.60	0.95, 2.81	0.0907	1.41	0.82, 2.53	0.2262
**Age group**								
18-44	66/90 (73.3%)	24/90 (26.7%)				ref	-	-
45-64	252/360 (70%)	108/360 (30%)				0.92	0.53, 1.54	0.7475
65+	170/282 (60.3%)	112/282 (39.7%)				0.61	0.35, 1.03	0.071
**Sex**								
Male	133/208 (63.9%)	75/208 (36.1%)				ref	-	-
Female	355/524 (67.7%)	169/524 (32.3%)				1.13	0.81, 1.59	0.4677
**Overall**	488/732 (66.7%)							

Table 23 Unadjusted odds ratio (OR) and adjusted odds ratio (aOR) for the association between ethnic minority status and experiencing limitations in self-care (n = 702).

	Experienced limitations	Did not experience limitations	Unadjusted model			Adjusted model		
	n/N (%)	n/N (%)	OR	95% CI	p-value	aOR	95% CI	p-value
**Minority ethnicity status**								
White British	156/623 (25.0%)	467/623 (75.0%)	ref	-	-	ref	-	-
Ethnic minority	26/79 (32.9%)	53/79 (67.1%)	1.47	0.88, 2.41	0.1344	1.27	0.74, 2.14	0.3735
**Age group**								
18-44	34/91 (37.4%)	57/91 (62.6%)				ref	-	-
45-64	81/343 (23.6%)	262/343 (76.4%)				0.54	0.33, 0.91	0.0186
65+	67/268 (25.0%)	201/268 (75.0%)				0.59	0.35, 1.01	0.0517
**Sex**								
Male	53/203 (26.1%)	150/203 (73.9%)				ref	-	-
Female	129/499 (25.9%)	370/499 (74.1%)				0.95	0.65, 1.39	0.7892
**Overall**	182/702 (25.9%)							

Table 24 Unadjusted odds ratio (OR) and adjusted odds ratio (aOR) for the association between ethnic minority status and experiencing limitations in taking care of others in the household (n = 586).

	**Experienced limitations**	**Did not experience limitations**	**Unadjusted model**			**Adjusted model**		
	**n/N (%)**	**n/N (%)**	**OR**	**95% CI**	**p-value**	**aOR**	**95% CI**	**p-value**
**Minority ethnicity status**								
White British	179/522 (34.3%)	343/522 (65.7%)	ref	-	-	ref	-	-
Ethnic minority	18/64 (28.1%)	46/64 (71.9%)	0.75	0.41, 1.31	0.3256	0.66	0.35, 1.17	0.1638
**Age group**								
18-44	33/79 (41.8%)	46/79 (58.2%)				ref	-	-
45-64	96/295 (32.5%)	199/295 (67.5%)				0.63	0.37, 1.06	0.0784
65+	68/212 (32.1%)	144/212 (67.9%)				0.61	0.35, 1.06	0.0757
**Sex**								
Male	54/173 (31.2%)	119/173 (68.8%)				ref	-	-
Female	143/413 (34.6%)	270/413 (65.4%)				1.17	0.80, 1.72	0.4297
**Overall**	197/586 (33.6%)							

Table 25 Unadjusted odds ratio (OR) and adjusted odds ratio (aOR) for the association between ethnic minority status and experiencing limitations in doing necessary activities in the house (n = 729).

	Experienced limitations	Did not experience limitations	Unadjusted model			Adjusted model		
	n/N (%)	n/N (%)	OR	95% CI	p-value	aOR	95% CI	p-value
**Minority ethnicity status**								
White British	397/650 (61.1%)	253/650 (38.9%)	ref	-	-	ref	-	-
Ethnic minority	45/79 (57.0%)	34/79 (43.0%)	0.84	0.53, 1.36	0.48	0.80	0.49, 1.31	0.3672
**Age group**								
18-44	56/90 (62.2%)	34/90 (37.8%)				ref	-	-
45-64	211/354 (59.6%)	143/354 (40.4%)				0.87	0.53, 1.41	0.5638
65+	175/285 (61.4%)	110/285 (38.6%)				0.94	0.56, 1.55	0.799
**Sex**								
Male	116/209 (55.5%)	93/209 (44.5%)				ref	-	-
Female	326/520 (62.7%)	194/520 (37.3%)				1.36	0.98, 1.89	0.0638
**Overall**	442/729 (60.6%)							

Table 26 Unadjusted odds ratio (OR) and adjusted odds ratio (aOR) for the association between ethnic minority status and experiencing limitations in doing enjoyable activities (n = 737).

	Experienced limitations	Did not experience limitations	Unadjusted model			Adjusted model		
	n/N (%)	n/N (%)	OR	95% CI	p-value	aOR	95% CI	p-value
**Minority ethnicity status**								
White British	479/658 (72.8%)	179/658 (27.2%)	ref	-	-	ref	-	-
Ethnic minority	57/79 (72.2%)	22/79 (27.8%)	0.97	0.58, 1.66	0.9033	0.92	0.54, 1.60	0.7502
**Age group**								
18-44	68/90 (75.6%)	22/90 (24.4%)				ref	-	-
45-64	254/356 (71.3%)	102/356 (28.7%)				0.8	0.45, 1.36	0.4185
65+	214/291 (73.5%)	77/291 (26.5%)				0.9	0.50, 1.56	0.7051
**Sex**								
Male	150/214 (70.1%)	64/214 (29.9%)				ref	-	-
Female	386/523 (73.8%)	137/523 (26.2%)				1.21	0.85, 1.72	0.2954
**Overall**	536/737 (72.7%)							

Sensitivity analyses 1 – Complete case analysis for IMD main analysis

Table 27 Sensitivity Analyses - Unadjusted odds ratio (OR) and adjusted odds ratio (aOR) for the association between IMD quintile and experiencing limitations in attending or participating in work or education (n = 396).

	Experienced limitations	Did not experience limitations	Unadjusted model			Adjusted model		
	n/N (%)	n/N (%)	OR	95% CI	p-value	aOR	95% CI	p-value
**IMD Quintile**								
1 (most deprived)	27/39 (69.2%)	12/39 (30.8%)	1.85	0.85, 4.21	0.1275	1.68	0.76, 3.86	0.2099
2	57/83 (68.7%)	26/83 (31.3%)	1.81	0.98, 3.38	0.061	1.66	0.89, 3.14	0.1166
3	55/88 (62.5%)	33/88 (37.5%)	1.37	0.76, 2.50	0.2962	1.30	0.71, 2.40	0.3958
4	55/93 (59.1%)	38/93 (40.9%)	1.19	0.67, 2.14	0.5537	1.25	0.68, 2.28	0.4729
5 (least deprived)	51/93 (54.8%)	42/93 (45.2%)	ref	-	-	ref	-	-
**Age group**								
18-44	49/72 (68.1%)	23/72 (31.9%)				ref	-	-
45-64	152/231 (65.8%)	79/231 (34.2%)				0.94	0.52, 1.66	0.8264
65+	44/93 (47.3%)	49/93 (52.7%)				0.47	0.24, 0.90	0.0248
**Sex**								
Male	72/118 (61.0%)	46/118 (39.0%)				ref	-	-
Female	173/278 (62.2%)	105/278 (37.8%)				0.98	0.62, 1.54	0.9292
**Migration status**								
UK Born	211/348 (60.6%)	137/348 (39.4%)				ref	-	-

Not UK Born	34/48 (70.8%)	14/48 (29.2%)				1.59	0.66, 3.96	0.3093
**Minority ethnicity status**								
White British	208/340 (61.2%)	132/340 (38.8%)				ref	-	-
Ethnic minority	37/56 (66.1%)	19/56 (33.9%)				0.80	0.35, 1.81	0.5854
**Overall**	245/396 (61.9%)							

Table 28 Sensitivity Analyses - Unadjusted odds ratio (OR) and adjusted odds ratio (aOR) for the association between IMD quintile and experiencing limitations in concentrating (n = 563).

	**Experienced limitations**	**Did not experience limitations**	**Unadjusted model**			**Adjusted model**		
	**n/N (%)**	**n/N (%)**	**OR**	**95% CI**	**p-value**	**aOR**	**95% CI**	**p-value**
**IMD Quintile**								
1 (most deprived)	42/51 (82.4%)	9/51 (17.6%)	3.22	1.52, 7.50	0.0038	2.89	1.35, 6.79	0.0094
2	76/106 (71.7%)	30/106 (28.3%)	1.75	1.03, 3.01	0.0411	1.62	0.95, 2.82	0.0793
3	84/125 (67.2%)	41/125 (32.8%)	1.41	0.86, 2.33	0.1733	1.31	0.79, 2.19	0.2880
4	81/134 (60.4%)	53/134 (39.6%)	1.05	0.65, 1.70	0.8291	1.03	0.63, 1.67	0.9179
5 (least deprived)	87/147 (59.2%)	60/147 (40.8%)	ref	-	-	ref	-	-
**Age group**								
18-44	50/71 (70.4%)	21/71 (29.6%)				ref	-	-
45-64	194/278 (69.8%)	84/278 (30.2%)				1.13	0.62, 2.03	0.6746
65+	126/214 (58.9%)	88/214 (41.1%)				0.77	0.41, 1.39	0.3883

**Sex**								
Male	100/163 (61.3%)	63/163 (38.7%)				ref	-	-
Female	270/400 (67.5%)	130/400 (32.5%)				1.25	0.85, 1.85	0.2596
**Migration status**								
UK Born	331/509 (65%)	178/509 (35%)				ref	-	-
Not UK Born	39/54 (72.2%)	15/54 (27.8%)				0.66	0.27, 1.60	0.3559
**Minority ethnicity status**								
White British	322/502 (64.1%)	180/502 (35.9%)				ref	-	-
Ethnic minority	48/61 (78.7%)	13/61 (21.3%)				2.25	0.95, 5.79	0.0773
**Overall**	370/563 (65.7%)							

Table 29 Sensitivity Analyses - Unadjusted odds ratio (OR) and adjusted odds ratio (aOR) for the association between IMD quintile and experiencing limitations in self-care (n = 538).

	**Experienced limitations**	**Did not experience limitations**	**Unadjusted model**			**Adjusted model**		
	**n/N (%)**	**n/N (%)**	**OR**	**95% CI**	**p-value**	**aOR**	**95% CI**	**p-value**
**IMD Quintile**								
1 (most deprived)	13/46 (28.3%)	33/46 (71.7%)	1.97	0.88, 4.27	0.0895	1.74	0.77, 3.85	0.1757
2	30/101 (29.7%)	71/101 (70.3%)	2.11	1.14, 3.95	0.0178	2.04	1.09, 3.85	0.0258
3	36/124 (29%)	88/124 (71%)	2.05	1.14, 3.74	0.0179	2.11	1.16, 3.89	0.0151
4	38/129 (29.5%)	91/129 (70.5%)	2.09	1.17, 3.79	0.0139	2.32	1.28, 4.27	0.0061

5 (least deprived)	23/138 (16.7%)	115/138 (83.3%)	ref	-	-	ref	-	-
**Age group**								
18-44	28/72 (38.9%)	44/72 (61.1%)				ref	-	-
45-64	64/264 (24.2%)	200/264 (75.8%)				0.51	0.29, 0.91	0.0214
65+	48/202 (23.8%)	154/202 (76.2%)				0.52	0.29, 0.97	0.0368
**Sex**								
Male	43/160 (26.9%)	117/160 (73.1%)				ref	-	
Female	97/378 (25.7%)	281/378 (74.3%)				0.82	0.53, 1.27	0.3667
**Migration status**								
UK Born	121/482 (25.1%)	361/482 (74.9%)				ref	-	-
Not UK Born	19/56 (33.9%)	37/56 (66.1%)				1.16	0.48, 2.73	0.7286
**Minority ethnicity status**								
White British	118/475 (24.8%)	357/475 (75.2%)				ref	-	-
Ethnic minority	22/63 (34.9%)	41/63 (65.1%)				1.32	0.57, 2.96	0.5102
**Overall**	140/538 (26.0%)							

Table 30 Sensitivity Analyses - Unadjusted odds ratio (OR) and adjusted odds ratio (aOR) for the association between IMD quintile and experiencing limitations in taking care of others in the household (n = 448).

	**Experienced limitations**	**Did not experience limitations**	**Unadjusted model**			**Adjusted model**		
	**n/N (%)**	**n/N (%)**	**OR**	**95% CI**	**p-value**	**aOR**	**95% CI**	**p-value**

**IMD Quintile**								
1 (most deprived)	16/40 (40.0%)	24/40 (60.0%)	1.48	0.70, 3.08	0.2927	1.41	0.66, 2.95	0.3676
2	26/79 (32.9%)	53/79 (67.1%)	1.09	0.60, 1.98	0.7747	1.05	0.57, 1.93	0.8651
3	31/96 (32.3%)	65/96 (67.7%)	1.06	0.60, 1.87	0.8376	1.05	0.59, 1.87	0.8683
4	39/104 (37.5%)	65/104 (62.5%)	1.33	0.77, 2.31	0.2986	1.27	0.73, 2.22	0.3993
5 (least deprived)	40/129 (31.0%)	89/129 (69.0%)	ref	-	-	ref	-	-
**Age group**								
18-44	27/63 (42.9%)	36/63 (57.1%)				ref	-	-
45-64	72/224 (32.1%)	152/224 (67.9%)				0.6	0.33, 1.08	0.0842
65+	53/161 (32.9%)	108/161 (67.1%)				0.62	0.33, 1.15	0.1293
**Sex**								
Male	41/136 (30.1%)	95/136 (69.9%)				ref	-	-
Female	111/312 (35.6%)	201/312 (64.4%)				1.24	0.80, 1.94	0.3412
**Migration status**								
UK Born	140/404 (34.7%)	264/404 (65.3%)				ref	-	-
Not UK Born	12/44 (27.3%)	32/44 (72.7%)				0.68	0.25, 1.76	0.4343
**Minority ethnicity status**								
White British	137/398 (34.4%)	261/398 (65.6%)				ref	-	-
Ethnic minority	15/50 (30.0%)	35/50 (70.0%)				0.95	0.38, 2.30	0.9119
**Overall**	152/448 (33.9%)							

Table 31 Sensitivity Analyses - Unadjusted odds ratio (OR) and adjusted odds ratio (aOR) for the association between IMD quintile and experiencing limitations in doing necessary activities outside the house (n = 562).

	Experienced limitations	Did not experience limitations	Unadjusted model			Adjusted model		
	n/N (%)	n/N (%)	OR	95% CI	p-value	aOR	95% CI	p-value
**IMD Quintile**								
1 (most deprived)	36/52 (69.2%)	16/52 (30.8%)	1.77	0.91, 3.55	0.0968	1.77	0.91, 3.58	0.1007
2	64/104 (61.5%)	40/104 (38.5%)	1.26	0.75, 2.12	0.3789	1.27	0.76, 2.15	0.3627
3	75/126 (59.5%)	51/126 (40.5%)	1.16	0.71, 1.88	0.5534	1.14	0.70, 1.86	0.6109
4	88/137 (64.2%)	49/137 (35.8%)	1.41	0.88, 2.29	0.1575	1.35	0.83, 2.21	0.223
5 (least deprived)	80/143 (55.9%)	63/143 (44.1%)	ref	-		ref	-	
**Age group**								
18-44	45/71 (63.4%)	26/71 (36.6%)				ref	-	-
45-64	163/273 (59.7%)	110/273 (40.3%)				0.86	0.49, 1.49	0.5952
65+	135/218 (61.9%)	83/218 (38.1%)				0.98	0.54, 1.75	0.9502
**Sex**								
Male	90/165 (54.5%)	75/165 (45.5%)				ref	-	-
Female	253/397 (63.7%)	144/397 (36.3%)				1.46	1.00, 2.12	0.0492
**Migration status**								
UK Born	309/507 (60.9%)	198/507 (39.1%)				ref	-	-

Not UK Born	34/55 (61.8%)	21/55 (38.2%)				1.32	0.59, 3.05	0.5048
**Minority ethnicity status**								
White British	306/499 (61.3%)	193/499 (38.7%)				ref	-	-
Ethnic minority	37/63 (58.7%)	26/63 (41.3%)				0.70	0.32, 1.52	0.3642
**Overall**	343/562 (61.0%)							

Table 32 Sensitivity Analyses - Unadjusted odds ratio (OR) and adjusted odds ratio (aOR) for the association between IMD quintile and experiencing limitations in doing enjoyable activities (n = 569).

	**Experienced limitations**	**Did not experience limitations**	**Unadjusted model**			**Adjusted model**		
	**n/N (%)**	**n/N (%)**	**OR**	**95% CI**	**p-value**	**aOR**	**95% CI**	**p-value**
**IMD Quintile**								
1 (most deprived)	41/51 (80.4%)	10/51 (19.6%)	1.87	0.89, 4.24	0.1139	1.90	0.90, 4.34	0.1076
2	77/107 (72%)	30/107 (28%)	1.17	0.68, 2.03	0.576	1.19	0.69, 2.08	0.5339
3	94/128 (73.4%)	34/128 (26.6%)	1.26	0.75, 2.14	0.3894	1.28	0.76, 2.18	0.3598
4	101/136 (74.3%)	35/136 (25.7%)	1.31	0.78, 2.22	0.302	1.33	0.79, 2.26	0.2876
5 (least deprived)	101/147 (68.7%)	46/147 (31.3%)	ref	-	-	ref	-	-
**Age group**								
18-44	53/71 (74.6%)	18/71 (25.4%)				ref	-	-
45-64	195/275 (70.9%)	80/275 (29.1%)				0.85	0.45, 1.54	0.5952
65+	166/223 (74.4%)	57/223 (25.6%)				1.05	0.54, 1.96	0.8858

**Sex**								
Male	123/171 (71.9%)	48/171 (28.1%)				ref	-	-
Female	291/398 (73.1%)	107/398 (26.9%)				1.05	0.70, 1.57	0.8119
**Migration status**								
UK Born	372/513 (72.5%)	141/513 (27.5%)				ref	-	-
Not UK Born	42/56 (75.0%)	14/56 (25.0%)				1.29	0.54, 3.20	0.5732
**Minority ethnicity status**								
White British	368/506 (72.7%)	138/506 (27.3%)				ref	-	-
Ethnic minority	46/63 (73.0%)	17/63 (27.0%)				0.84	0.37, 1.96	0.6778
**Overall**	414/569 (72.8%)							

Sensitivity analyses 2 – IMD quintile (without the health domain) as exposure

Table 33 Sensitivity Analyses - Unadjusted odds ratio (OR) and adjusted odds ratio (aOR) for the association between IMD quintile (without the health domain) and experiencing limitations in attending or participating in work or education (n = 516).

	**Experienced limitations**	**Did not experience limitations**	**Unadjusted model**			**Adjusted model**		
	**n/N (%)**	**n/N (%)**	**OR**	**95% CI**	**p-value**	**aOR**	**95% CI**	**p-value**
**IMD Quintile**								
1 (most deprived)	36/50 (72%)	14/50 (28%)	2.31	1.15, 4.87	0.0221	2.11	1.04, 4.48	0.0439
2	77/106 (72.6%)	29/106 (27.4%)	2.39	1.37, 4.23	0.0024	2.24	1.27, 4.00	0.0058
3	74/127 (58.3%)	53/127 (41.7%)	1.26	0.76, 2.10	0.3795	1.24	0.74, 2.09	0.4119

4	67/119 (56.3%)	52/119 (43.7%)	1.16	0.69, 1.95	0.5739	1.15	0.68, 1.94	0.6131
5 (least deprived)	60/114 (52.6%)	54/114 (47.4%)	ref	-	-	ref	-	-
**Age group**								
18-44	60/90 (66.7%)	30/90 (33.3%)				ref	-	-
45-64	189/293 (64.5%)	104/293 (35.5%)				0.93	0.55, 1.56	0.7902
65+	65/133 (48.9%)	68/133 (51.1%)				0.53	0.29, 0.94	0.031
**Sex**								
Male	90/148 (60.8%)	58/148 (39.2%)				ref	-	-
Female	224/368 (60.9%)	144/368 (39.1%)				0.98	0.65, 1.46	0.9121
**Migration status**								
UK Born	211/348 (60.6%)	137/348 (39.4%)				ref	-	-
Not UK Born	34/48 (70.8%)	14/48 (29.2%)				1.51	0.67, 3.52	0.3285
Missing	69/120 (57.5%)	51/120 (42.5%)				0.94	0.61, 1.46	0.787
**Minority ethnicity status**								
White British	270/447 (60.4%)	177/447 (39.6%)				ref	-	-
Ethnic minority	44/69 (63.8%)	25/69 (36.2%)				0.78	0.39, 1.55	0.4698
**Overall**	314/516 (60.9%)							

Table 34 Sensitivity Analyses - Unadjusted odds ratio (OR) and adjusted odds ratio (aOR) for the association between IMD quintile (without the health domain) and experiencing limitations in concentrating (n = 727).

	Experienced limitations	Did not experience limitations	Unadjusted model			Adjusted model		
	n/N (%)	n/N (%)	OR	95% CI	p-value	aOR	95% CI	p-value
**IMD Quintile**								
1 (most deprived)	53/65 (81.5%)	12/65 (18.5%)	2.97	1.52, 6.20	0.0022	2.80	1.43, 5.87	0.0041
2	98/132 (74.2%)	34/132 (25.8%)	1.94	1.19, 3.20	0.0086	1.87	1.14, 3.10	0.0144
3	116/174 (66.7%)	58/174 (33.3%)	1.35	0.87, 2.09	0.1827	1.34	0.86, 2.09	0.1929
4	113/182 (62.1%)	69/182 (37.9%)	1.10	0.72, 1.69	0.6541	1.13	0.73, 1.75	0.5728
5 (least deprived)	104/174 (59.8%)	70/174 (40.2%)	ref	-	-	ref	-	-
**Age group**								
18-44	65/89 (73%)	24/89 (27%)				ref	-	-
45-64	249/357 (69.7%)	108/357 (30.3%)				0.96	0.55, 1.62	0.8686
65+	170/281 (60.5%)	111/281 (39.5%)				0.68	0.38, 1.16	0.1608
**Sex**								
Male	132/207 (63.8%)	75/207 (36.2%)				ref	-	-
Female	352/520 (67.7%)	168/520 (32.3%)				1.12	0.79, 1.58	0.5155
**Migration status**								
UK Born	331/509 (65%)	178/509 (35%)				ref	-	-
Not UK Born	39/54 (72.2%)	15/54 (27.8%)				0.88	0.40, 1.98	0.7575

Missing	114/164 (69.5%)	50/164 (30.5%)				1.24	0.84, 1.83	0.2847
**Minority ethnicity status**								
White British	426/650 (65.5%)	224/650 (34.5%)				ref	-	-
Ethnic minority	58/77 (75.3%)	19/77 (24.7%)				1.42	0.72, 2.93	0.3286
**Overall**	484/727 (66.6%)							

Table 35 Sensitivity Analyses - Unadjusted odds ratio (OR) and adjusted odds ratio (aOR) for the association between IMD quintile (without the health domain) and experiencing limitations in self-care (n = 697).

	**Experienced limitations**	**Did not experience limitations**	**Unadjusted model**			**Adjusted model**		
	**n/N (%)**	**n/N (%)**	**OR**	**95% CI**	**p-value**	**aOR**	**95% CI**	**p-value**
**IMD Quintile**								
1 (most deprived)	22/59 (37.3%)	37/59 (62.7%)	2.99	1.53, 5.87	0.0013	2.79	1.41, 5.53	0.0031
2	35/127 (27.6%)	92/127 (72.4%)	1.92	1.09, 3.40	0.0247	1.85	1.04, 3.30	0.0369
3	38/169 (22.5%)	131/169 (77.5%)	1.46	0.85, 2.55	0.1755	1.48	0.85, 2.59	0.1652
4	58/179 (32.4%)	121/179 (67.6%)	2.41	1.45, 4.10	0.0009	2.49	1.49, 4.26	0.0006
5 (least deprived)	27/163 (16.6%)	136/163 (83.4%)	ref	-	-	ref	-	-
**Age group**								
18-44	33/90 (36.7%)	57/90 (63.3%)				ref	-	-
45-64	80/340 (23.5%)	260/340 (76.5%)				0.59	0.35, 0.99	0.0435
65+	67/267 (25.1%)	200/267 (74.9%)				0.66	0.39, 1.15	0.1369

**Sex**								
Male	52/202 (25.7%)	150/202 (74.3%)				ref	-	-
Female	128/495 (25.9%)	367/495 (74.1%)				0.92	0.63, 1.36	0.6774
**Migration status**								
UK Born	121/482 (25.1%)	361/482 (74.9%)				ref	-	-
Not UK Born	19/56 (33.9%)	37/56 (66.1%)				1.25	0.56, 2.72	0.5735
Missing	40/159 (25.2%)	119/159 (74.8%)				1.05	0.68, 1.59	0.8214
**Minority ethnicity status**								
White British	154/618 (24.9%)	464/618 (75.1%)				ref	-	-
Ethnic minority	26/79 (32.9%)	53/79 (67.1%)				1.20	0.59, 2.35	0.6048
**Overall**	180/697 (25.8%)							

Table 36 Sensitivity Analyses - Unadjusted odds ratio (OR) and adjusted odds ratio (aOR) for the association between IMD quintile (without the health domain) and experiencing limitations in taking care of others in the household (n = 581).

	**Experienced limitations**	**Did not experience limitations**	**Unadjusted model**			**Adjusted model**		
	**n/N (%)**	**n/N (%)**	**OR**	**95% CI**	**p-value**	**aOR**	**95% CI**	**p-value**
**IMD Quintile**								
1 (most deprived)	21/50 (42.0%)	29/50 (58.0%)	1.81	0.93, 3.50	0.0787	1.75	0.89, 3.42	0.1006
2	38/99 (38.4%)	61/99 (61.6%)	1.56	0.91, 2.66	0.1046	1.53	0.89, 2.63	0.1261
3	35/132 (26.5%)	97/132 (73.5%)	0.90	0.53, 1.52	0.6983	0.87	0.51, 1.47	0.6011
4	57/146	89/146	1.60	0.99, 2.60	0.0559	1.51	0.93, 2.47	0.0982

	(39.0%)	(61.0%)							
5 (least deprived)	44/154 (28.6%)	110/154 (71.4%)	ref	-	-	ref	-	-
**Age group**									
18-44	32/78 (41.0%)	46/78 (59.0%)				ref	-	-
45-64	95/292 (32.5%)	197/292 (67.5%)				0.67	0.39, 1.14	0.1371
65+	68/211 (32.2%)	143/211 (67.8%)				0.66	0.38, 1.17	0.153
**Sex**									
Male	53/172 (30.8%)	119/172 (69.2%)				ref	-	-
Female	142/409 (34.7%)	267/409 (65.3%)				1.17	0.79, 1.73	0.4348
**Migration status**									
UK Born	140/404 (34.7%)	264/404 (65.3%)				ref	-	-
Not UK Born	12/44 (27.3%)	32/44 (72.7%)				0.76	0.30, 1.81	0.5362
Missing	43/133 (32.3%)	90/133 (67.7%)				0.94	0.61, 1.44	0.7904
**Minority ethnicity status**									
White British	177/517 (34.2%)	340/517 (65.8%)				ref	-	-
Ethnic minority	18/64 (28.1%)	46/64 (71.9%)				0.77	0.36, 1.58	0.4834
**Overall**	195/581 (33.6%)								

Table 37 Sensitivity Analyses - Unadjusted odds ratio (OR) and adjusted odds ratio (aOR) for the association between IMD quintile (without the health domain) and experiencing limitations in doing necessary activities outside the house (n = 724).

	Experienced limitations	Did not experience limitations	Unadjusted model			Adjusted model		
	n/N (%)	n/N (%)	OR	95% CI	p-value	aOR	95% CI	p-value
**IMD Quintile**								
1 (most deprived)	45/64 (70.3%)	19/64 (29.7%)	2.03	1.11, 3.82	0.0242	2.03	1.10, 3.85	0.0259
2	88/130 (67.7%)	42/130 (32.3%)	1.80	1.12, 2.91	0.0159	1.79	1.11, 2.91	0.0181
3	97/175 (55.4%)	78/175 (44.6%)	1.07	0.70, 1.63	0.7682	1.04	0.68, 1.59	0.8723
4	117/186 (62.9%)	69/186 (37.1%)	1.45	0.95, 2.23	0.0841	1.39	0.91, 2.14	0.1322
5 (least deprived)	91/169 (53.8%)	78/169 (46.2%)	ref	-	-	ref	-	-
**Age group**								
18-44	55/89 (61.8%)	34/89 (38.2%)				ref	-	-
45-64	209/351 (59.5%)	142/351 (40.5%)				0.92	0.55, 1.50	0.7304
65+	174/284 (61.3%)	110/284 (38.7%)				1.03	0.61, 1.72	0.9108
**Sex**								
Male	115/208 (55.3%)	93/208 (44.7%)				ref	-	-
Female	323/516 (62.6%)	193/516 (37.4%)				1.36	0.98, 1.90	0.0664
**Migration status**								
UK Born	309/507 (60.9%)	198/507 (39.1%)				ref	-	-
Not UK Born	34/55 (61.8%)	21/55 (38.2%)				1.27	0.61, 2.70	0.5315

Missing	95/162 (58.6%)	67/162 (41.4%)				0.95	0.66, 1.38	0.7996
**Minority ethnicity status**								
White British	393/645 (60.9%)	252/645 (39.1%)				ref	-	-
Ethnic minority	45/79 (57.0%)	34/79 (43.0%)				0.68	0.36, 1.28	0.2317
**Overall**	438/724 (60.5%)							

Table 38 Sensitivity Analyses - Unadjusted odds ratio (OR) and adjusted odds ratio (aOR) for the association between IMD quintile (without the health domain) and experiencing limitations in doing enjoyable activities (n = 732).

	**Experienced limitations**	**Did not experience limitations**	**Unadjusted model**			**Adjusted model**		
	**n/N (%)**	**n/N (%)**	**OR**	**95% CI**	**p-value**	**aOR**	**95% CI**	**p-value**
**IMD Quintile**								
1 (most deprived)	51/63 (81%)	12/63 (19%)	2.05	1.04, 4.31	0.0452	2.04	1.03, 4.30	0.0496
2	99/133 (74.4%)	34/133 (25.6%)	1.41	0.86, 2.34	0.1827	1.40	0.84, 2.33	0.1968
3	122/173 (70.5%)	51/173 (29.5%)	1.16	0.73, 1.82	0.5332	1.14	0.73, 1.81	0.5622
4	142/188 (75.5%)	46/188 (24.5%)	1.49	0.94, 2.37	0.0879	1.47	0.92, 2.34	0.1046
5 (least deprived)	118/175 (67.4%)	57/175 (32.6%)	ref	-	-	ref	-	-
**Age group**								
18-44	67/89 (75.3%)	22/89 (24.7%)				ref	-	-
45-64	252/353 (71.4%)	101/353 (28.6%)				0.84	0.48, 1.45	0.5486
65+	213/290 (73.4%)	77/290 (26.6%)				0.96	0.53, 1.69	0.9007

**Sex**								
Male	149/213 (70%)	64/213 (30%)				ref	-	-
Female	383/519 (73.8%)	136/519 (26.2%)				1.20	0.84, 1.71	0.3143
**Migration status**								
UK Born	372/513 (72.5%)	141/513 (27.5%)				ref	-	-
Not UK Born	42/56 (75%)	14/56 (25%)				1.26	0.57, 2.90	0.5703
Missing	118/163 (72.4%)	45/163 (27.6%)				1.03	0.69, 1.55	0.8784
**Minority ethnicity status**								
White British	475/653 (72.7%)	178/653 (27.3%)				ref	-	-
Ethnic minority	57/79 (72.2%)	22/79 (27.8%)				0.81	0.42, 1.63	0.5487
**Overall**	532/732 (72.7%)							

Sensitivity Analyses 3 – Migration status with the “Missing” category as the exposure

Table 39 Sensitivity Analyses - Unadjusted odds ratio (OR) and adjusted odds ratio (aOR) for the association between migration status (including the “Missing” category) and experiencing limitations in attending or participating in work or education (n = 520).

	**Experienced limitations**	**Did not experience limitations**	**Unadjusted model**			**Adjusted model**		
	**n/N (%)**	**n/N (%)**	**OR**	**95% CI**	**p-value**	**aOR**	**95% CI**	**p-value**
**Migration status**								
UK Born	215/352 (61.1%)	137/352 (38.9%)	ref	-	-	ref	-	-
Not UK Born	34/48 (70.8%)	14/48 (29.2%)	1.55	0.82, 3.08	0.1936	1.6	0.72, 3.72	0.2584
Missing	69/120 (57.5%)	51/120 (42.5%)	0.86	0.57, 1.32	0.4893	0.92	0.60, 1.41	0.692
**Age group**								
18-44	61/91 (67%)	30/91 (33%)				ref	-	-
45-64	192/296 (64.9%)	104/296 (35.1%)				0.91	0.54, 1.51	0.7196
65+	65/133 (48.9%)	68/133 (51.1%)				0.48	0.27, 0.85	0.0128
**Sex**								
Male	91/149 (61.1%)	58/149 (38.9%)				ref	-	-
Female	227/371 (61.2%)	144/371 (38.8%)				1.01	0.68, 1.50	0.9606
**Minority Ethnicity Status**								
White British	274/451 (60.8%)	177/451 (39.2%)				ref	-	-
Ethnic minority	44/69 (63.8%)	25/69 (36.2%)				0.8	0.40, 1.57	0.5067
**Overall**	318/520 (61.2%)							

Table 40 Sensitivity Analyses - Unadjusted odds ratio (OR) and adjusted odds ratio (aOR) for the association between migration status (including the "Missing" category) and experiencing limitations in concentrating (n = 732).

	Experienced limitations	Did not experience limitations	Unadjusted model			Adjusted model		
	n/N (%)	n/N (%)	OR	95% CI	p-value	aOR	95% CI	p-value
**Migration status**								
UK Born	335/514 (65.2%)	179/514 (34.8%)	ref	-	-	ref	-	
Not UK Born	39/54 (72.2%)	15/54 (27.8%)	1.39	0.76, 2.66	0.3006	0.95	0.44, 2.13	0.9068
Missing	114/164 (69.5%)	50/164 (30.5%)	1.22	0.84, 1.79	0.3069	1.20	0.82, 1.77	0.3538
**Age group**								
18-44	66/90 (73.3%)	24/90 (26.7%)				ref	-	
45-64	252/360 (70%)	108/360 (30%)				0.91	0.53, 1.54	0.7359
65+	170/282 (60.3%)	112/282 (39.7%)				0.60	0.35, 1.02	0.0664
**Sex**								
Male	133/208 (63.9%)	75/208 (36.1%)				ref	-	
Female	355/524 (67.7%)	169/524 (32.3%)				1.13	0.80, 1.59	0.4824
**Minority Ethnicity Status**								
White British	430/655 (65.6%)	225/655 (34.4%)				ref	-	
Ethnic minority	58/77 (75.3%)	19/77 (24.7%)				1.45	0.74, 3.00	0.2934
**Overall**	488/732 (66.7%)							

Table 41 Sensitivity Analyses - Unadjusted odds ratio (OR) and adjusted odds ratio (aOR) for the association between migration status (including the "Missing" category) and experiencing limitations in self-care (n = 702).

	Experienced limitations	Did not experience limitations	Unadjusted model			Adjusted model		
	n/N (%)	n/N (%)	OR	95% CI	p-value	aOR	95% CI	p-value
**Migration status**	123/487 (25.3%)	364/487 (74.7%)						
UK Born	19/56 (33.9%)	37/56 (66.1%)	ref	-	-	ref	-	-
Not UK Born	40/159 (25.2%)	119/159 (74.8%)	1.52	0.83, 2.71	0.1643	1.27	0.58, 2.73	0.5369
Missing			0.99	0.65, 1.49	0.98	0.99	0.65, 1.49	0.9593
**Age group**	34/91 (37.4%)	57/91 (62.6%)						
18-44	81/343 (23.6%)	262/343 (76.4%)				ref	-	-
45-64	67/268 (25%)	201/268 (75%)				0.54	0.33, 0.91	0.0181
65+						0.59	0.35, 1.01	0.0535
**Sex**	53/203 (26.1%)	150/203 (73.9%)						
Male	129/499 (25.9%)	370/499 (74.1%)				ref	-	-
Female						0.95	0.66, 1.39	0.7995
**Minority Ethnicity Status**	156/623 (25%)	467/623 (75%)						
White British	26/79 (32.9%)	53/79 (67.1%)				ref	-	-
Ethnic minority	182/702 (25.9%)					1.12	0.56, 2.16	0.7466
**Overall**	123/487 (25.3%)	364/487 (74.7%)						

Table 42 Sensitivity Analyses - Unadjusted odds ratio (OR) and adjusted odds ratio (aOR) for the association between migration status (including the "Missing" category) and experiencing limitations in taking care of others in the household (n = 586).

	Experienced limitations	Did not experience limitations	Unadjusted model			Adjusted model		
	n/N (%)	n/N (%)	OR	95% CI	p-value	aOR	95% CI	p-value
**Migration status**								
UK Born	142/409 (34.7%)	267/409 (65.3%)	ref	-	-	ref	-	-
Not UK Born	12/44 (27.3%)	32/44 (72.7%)	0.71	0.34, 1.38	0.3237	0.79	0.32, 1.87	0.5967
Missing	43/133 (32.3%)	90/133 (67.7%)	0.90	0.59, 1.36	0.614	0.92	0.60, 1.39	0.6825
**Age group**								
18-44	33/79 (41.8%)	46/79 (58.2%)				ref	-	-
45-64	96/295 (32.5%)	199/295 (67.5%)				0.63	0.37, 1.06	0.0798
65+	68/212 (32.1%)	144/212 (67.9%)				0.61	0.35, 1.05	0.0741
**Sex**								
Male	54/173 (31.2%)	119/173 (68.8%)				ref	-	-
Female	143/413 (34.6%)	270/413 (65.4%)				1.17	0.80, 1.72	0.4338
**Minority Ethnicity Status**								
White British	179/522 (34.3%)	343/522 (65.7%)				ref	-	-
Ethnic minority	18/64 (28.1%)	46/64 (71.9%)				0.74	0.35, 1.51	0.4166
**Overall**	197/586 (33.6%)							

Table 43 Sensitivity Analyses - Unadjusted odds ratio (OR) and adjusted odds ratio (aOR) for the association between migration status (including the "Missing" category) and experiencing limitations in doing necessary activities outside the house (n = 729).

	Experienced limitations	Did not experience limitations	Unadjusted model			Adjusted model		
	n/N (%)	n/N (%)	OR	95% CI	p-value	aOR	95% CI	p-value
**Migration status**								
UK Born	313/512 (61.1%)	199/512 (38.9%)	ref	-	-	ref	-	-
Not UK Born	34/55 (61.8%)	21/55 (38.2%)	1.03	0.59, 1.85	0.921	1.32	0.64, 2.81	0.4576
Missing	95/162 (58.6%)	67/162 (41.4%)	0.90	0.63, 1.29	0.5719	0.91	0.63, 1.31	0.6159
**Age group**								
18-44	56/90 (62.2%)	34/90 (37.8%)				ref	-	-
45-64	211/354 (59.6%)	143/354 (40.4%)				0.86	0.53, 1.41	0.5623
65+	175/285 (61.4%)	110/285 (38.6%)				0.94	0.56, 1.56	0.8172
**Sex**								
Male	116/209 (55.5%)	93/209 (44.5%)				ref	-	-
Female	326/520 (62.7%)	194/520 (37.3%)				1.37	0.99, 1.90	0.0596
**Minority Ethnicity Status**								
White British	397/650 (61.1%)	253/650 (38.9%)				ref	-	-
Ethnic minority	45/79 (57%)	34/79 (43%)				0.69	0.37, 1.29	0.2395
**Overall**	442/729 (60.6%)							

Table 44 Sensitivity Analyses - Unadjusted odds ratio (OR) and adjusted odds ratio (aOR) for the association between migration status (including the "Missing" category) and experiencing limitations in doing enjoyable activities (n = 737).

	**Experienced limitations**	**Did not experience limitations**	**Unadjusted model**			**Adjusted model**		
	**n/N (%)**	**n/N (%)**	**OR**	**95% CI**	**p-value**	**aOR**	**95% CI**	**p-value**
**Migration status**								
UK Born	376/518 (72.6%)	142/518 (27.4%)	ref	-	-	ref	-	-
Not UK Born	42/56 (75.0%)	14/56 (25.0%)	1.13	0.61, 2.21	0.6999	1.29	0.59, 2.95	0.5313
Missing	118/163 (72.4%)	45/163 (27.6%)	0.99	0.67, 1.48	0.9614	0.99	0.67, 1.49	0.9728
**Age group**								
18-44	68/90 (75.6%)	22/90 (24.4%)				ref	-	-
45-64	254/356 (71.3%)	102/356 (28.7%)				0.80	0.45, 1.36	0.4139
65+	214/291 (73.5%)	77/291 (26.5%)				0.90	0.50, 1.57	0.7142
**Sex**								
Male	150/214 (70.1%)	64/214 (29.9%)				ref	-	-
Female	386/523 (73.8%)	137/523 (26.2%)				1.21	0.85, 1.72	0.29
**Minority Ethnicity Status**								
White British	479/658 (72.8%)	179/658 (27.2%)				ref	-	-
Ethnic minority	57/79 (72.2%)	22/79 (27.8%)				0.80	0.41, 1.60	0.5235
**Overall**	536/737 (72.7%)							

Virus Watch Long-term Symptoms Survey Example

Long-Term Symptoms

In the past six months (i.e., since October 2023), have any of the following household members experienced any persistent symptoms that have lasted for four or more weeks (even if these symptoms come and go) and that are not explained by something else (e.g. pre-existing chronic illness or pregnancy)?

	Yes	No	Prefer not to say
[go_arm_1][hh1_fname] [go_arm_1][hh1_sname]	○	○	○
[go_arm_1][hh2_fname] [go_arm_1][hh2_sname]	○	○	○
[go_arm_1][hh3_fname] [go_arm_1][hh3_sname]	○	○	○
[go_arm_1][hh4_fname] [go_arm_1][hh4_sname]	○	○	○
[go_arm_1][hh5_fname] [go_arm_1][hh5_sname]	○	○	○
[go_arm_1][hh6_fname] [go_arm_1][hh6_sname]	○	○	○

Long-Term Symptoms for [go_arm_1][hh1_fname] [go_arm_1][hh1_sname]

[go_arm_1][hh1_fname] [go_arm_1][hh1_sname]: Please pick all of the persistent symptoms that you have experienced for four or more weeks.

These are new symptoms that you experienced for four or more weeks in the last last six months (i.e., since October 2023) and that are not explained by something else (e.g. pre-existing chronic illness or pregnancy). These symptoms could be constant or could come and go.

Fever	☐
Unintentional weight loss	☐
Unintentional weight gain	☐
Sleep problems (including difficulty falling asleep, staying asleep, oversleeping, or sleep apnoea)	☐
Change in appetite	☐
Fatigue	☐
Post-exertion malaise (feeling unwell or 'crashing' after exercise or physical exertion)	☐
Headache	☐
Dizziness	☐
Problems with movement, balance, and/or co-ordination	☐
Memory problems	☐
Brain fog and/or other cognitive problems	☐
Paraesthesia (pricking, tingling, or creeping feeling on the skin)	☐
Change/loss of taste	☐
Change/loss of smell	☐
Feeling anxious	☐
Feeling depressed	☐
Hair loss	☐
Skin rash	☐
Swelling of hands and/or feet	☐
Dry eyes / redness of eyes	☐
Blurry vision	☐
Visual changes	☐
Sore throat or mouth	☐
Ear pain	☐
Change in hearing	☐
Congested / runny nose	☐

Difficulty swallowing (dysphagia)	☐
Tinnitus (ringing or other noises in one or both ears)	☐
Chest pain	☐
Shortness of breath / dyspnoea	☐
Cough	☐
Tachycardia (fast heartbeat) or palpitations	☐
Easy bruising / bleeding	☐
Swollen lymph nodes	☐
Abdominal pain	☐
Nausea and/or vomiting	☐
Diarrhoea	☐
Acid reflux / heartburn	☐
Gynaecological problems (e.g. change in menstrual cycles or flow)	☐
Urinary symptoms (e.g. bladder frequency, urgency, or incontinence)	☐
Bone and/or joint pain	☐
Muscle aches	☐
New allergy (e.g. to food, medication, etc.)	☐
Other symptom(s), please describe below	☐

If other, please describe: ______________________________

Most Severe Symptoms

Think about up to three of these symptoms that were the worst or that gave you the most trouble.

Please select one of these severe symptoms (these don't have to be in order). If only one symptom caused you the most trouble than please select this one.

- Fever ○
- Unintentional weight loss ○
- Unintentional weight gain ○
- Sleep problems (including difficulty falling asleep, staying asleep, oversleeping, or sleep apnoea) ○
- Change in appetite ○
- Fatigue ○
- Post-exertion malaise (feeling unwell or 'crashing' after exercise or physical exertion) ○
- Headache ○
- Dizziness ○
- Problems with movement, balance, and/or co-ordination ○
- Memory problems ○
- Brain fog and/or other cognitive problems ○
- Paraesthesia (pricking, tingling, or creeping feeling on the skin) ○
- Change/loss of taste ○
- Change/loss of smell ○
- Feeling anxious ○
- Feeling depressed ○
- Hair loss ○
- Skin rash ○
- Swelling of hands and/or feet ○
- Dry eyes / redness of eyes ○
- Blurry vision ○
- Visual changes ○
- Sore throat or mouth ○
- Ear pain ○
- Change in hearing ○
- Congested / runny nose ○
- Difficulty swallowing (dysphagia) ○

Tinnitus (ringing or other noises in one or both ears)	◯
Chest pain	◯
Shortness of breath / dyspnoea	◯
Cough	◯
Tachycardia (fast heartbeat) or palpitations	◯
Easy bruising / bleeding	◯
Swollen lymph nodes	◯
Abdominal pain	◯
Nausea and/or vomiting	◯
Diarrhoea	◯
Acid reflux / heartburn	◯
Gynaecological problems (e.g. change in menstrual cycles or flow)	◯
Urinary symptoms (e.g. bladder frequency, urgency, or incontinence)	◯
Bone and/or joint pain	◯
Muscle aches	◯
New allergy (e.g. to food, medication, etc.)	◯
Other symptom(s), please describe below	◯

If other, please describe: ______________________

Approximately when did this symptom first appear? ______________________

Please provide your best guess if you cannot remember.

Is this symptom still ongoing?
◯ Yes
◯ No

Approximately when did this symptom stop? ______________________

Please provide your best guess if you cannot remember.

Please describe the overall frequency of this symptom. If the symptom is no longer ongoing, please describe its frequency when you used to experience the symptom.
◯ Continuous (every day)
◯ A few times a week
◯ Less than weekly

Which of the following best describes how you experience(d) this symptom?
◯ Consistent in its frequency and severity
◯ Fluctuating (changes from time to time in how frequent or how severe it is)
◯ Relapsing (improved or disappeared for a while and then returned or became severe again)

If more than one symptom gave you lots of trouble, please select the next symptom.

If only one symptom was the worst, please select N/A

N/A	○
Fever	○
Unintentional weight loss	○
Unintentional weight gain	○
Sleep problems (including difficulty falling asleep, staying asleep, oversleeping, or sleep apnoea)	○
Change in appetite	○
Fatigue	○
Post-exertion malaise (feeling unwell or 'crashing' after exercise or physical exertion)	○
Headache	○
Dizziness	○
Problems with movement, balance, and/or co-ordination	○
Memory problems	○
Brain fog and/or other cognitive problems	○
Paraesthesia (pricking, tingling, or creeping feeling on the skin)	○
Change/loss of taste	○
Change/loss of smell	○
Feeling anxious	○
Feeling depressed	○
Hair loss	○
Skin rash	○
Swelling of hands and/or feet	○
Dry eyes / redness of eyes	○
Blurry vision	○
Visual changes	○
Sore throat or mouth	○
Ear pain	○
Change in hearing	○
Congested / runny nose	○
Difficulty swallowing (dysphagia)	○

Tinnitus (ringing or other noises in one or both ears)	◯
Chest pain	◯
Shortness of breath / dyspnoea	◯
Cough	◯
Tachycardia (fast heartbeat) or palpitations	◯
Easy bruising / bleeding	◯
Swollen lymph nodes	◯
Abdominal pain	◯
Nausea and/or vomiting	◯
Diarrhoea	◯
Acid reflux / heartburn	◯
Gynaecological problems (e.g. change in menstrual cycles or flow)	◯
Urinary symptoms (e.g. bladder frequency, urgency, or incontinence)	◯
Bone and/or joint pain	◯
Muscle aches	◯
New allergy (e.g. to food, medication, etc.)	◯
Other symptom(s), please describe below	◯

If other, please describe: ________________________________

Approximately when did this symptom first appear? ________________________________

Please provide your best guess if you cannot remember.

Is this symptom still ongoing?
- ◯ Yes
- ◯ No

Approximately when did this symptom stop? ________________________________

Please provide your best guess if you cannot remember.

Please describe the overall frequency of this symptom.
If the symptom is no longer ongoing, please describe its frequency when you used to experience the symptom.
- ◯ Continuous (every day)
- ◯ A few times a week
- ◯ Less than weekly

Which of the following best describes how you experience(d) this symptom?
- ◯ Consistent in its frequency and severity
- ◯ Fluctuating (changes from time to time in how frequent or how severe it is)
- ◯ Relapsing (improved or disappeared for a while and then returned or became severe again)

If a third symptom gave you lots of trouble, please select the next symptom.

If not, please select N/A

N/A	○
Fever	○
Unintentional weight loss	○
Unintentional weight gain	○
Sleep problems (including difficulty falling asleep, staying asleep, oversleeping, or sleep apnoea)	○
Change in appetite	○
Fatigue	○
Post-exertion malaise (feeling unwell or 'crashing' after exercise or physical exertion)	○
Headache	○
Dizziness	○
Problems with movement, balance, and/or co-ordination	○
Memory problems	○
Brain fog and/or other cognitive problems	○
Paraesthesia (pricking, tingling, or creeping feeling on the skin)	○
Change/loss of taste	○
Change/loss of smell	○
Feeling anxious	○
Feeling depressed	○
Hair loss	○
Skin rash	○
Swelling of hands and/or feet	○
Dry eyes / redness of eyes	○
Blurry vision	○
Visual changes	○
Sore throat or mouth	○
Ear pain	○
Change in hearing	○
Congested / runny nose	○
Difficulty swallowing (dysphagia)	○
Tinnitus (ringing or other noises in one or both ears)	○
Chest pain	○
Shortness of breath / dyspnoea	○

Cough	◯
Tachycardia (fast heartbeat) or palpitations	◯
Easy bruising / bleeding	◯
Swollen lymph nodes	◯
Abdominal pain	◯
Nausea and/or vomiting	◯
Diarrhoea	◯
Acid reflux / heartburn	◯
Gynaecological problems (e.g. change in menstrual cycles or flow)	◯
Urinary symptoms (e.g. bladder frequency, urgency, or incontinence)	◯
Bone and/or joint pain	◯
Muscle aches	◯
New allergy (e.g. to food, medication, etc.)	◯
Other symptom(s), please describe below	◯

If other, please describe: ________________________________

Approximately when did this symptom first appear? ________________________________

Please provide your best guess if you cannot remember.

Is this symptom still ongoing?
- ◯ Yes
- ◯ No

Approximately when did this symptom stop? ________________________________

Please provide your best guess if you cannot remember.

Please describe the overall frequency of this symptom.
If the symptom is no longer ongoing, please describe
its frequency when you used to experience the symptom.
- ◯ Continuous (every day)
- ◯ A few times a week
- ◯ Less than weekly

Which of the following best describes how you experience(d) this symptom?
- ◯ Consistent in its frequency and severity
- ◯ Fluctuating (changes from time to time in how frequent or how severe it is)
- ◯ Relapsing (improved or disappeared for a while and then returned or became severe again)

Overall Impact of Symptoms

Did your symptoms make it more difficult to:

	Yes, a lot	Yes, a little	Not at all	N/A
Go to/participate in work or education	◯	◯	◯	◯
Concentrate on things (e.g. reading, watching TV)	◯	◯	◯	◯
Take care of yourself (e.g. wash, dress, and feed yourself)	◯	◯	◯	◯
Take care of others in the household	◯	◯	◯	◯
Do necessary daily activities outside the house (e.g. shopping)	◯	◯	◯	◯
Do activities that you enjoy (e.g. hobbies)	◯	◯	◯	◯

Did these long-term symptoms follow an initial short-term (acute) illness, e.g. a COVID or flu-like illness or a gastrointestinal illness?

◯ Yes
◯ No
◯ Unsure

Do you think these symptoms were due to COVID (i.e. you have or had long-COVID)?

◯ Yes
◯ No
◯ Unsure

Optional: Please describe your symptoms in your own words

____

Optional: Please describe your the impact these symptoms have had on you and/or your family

____
